# Reliable Facility Location Problem with Facility Protection

**DOI:** 10.1371/journal.pone.0161532

**Published:** 2016-09-01

**Authors:** Luohao Tang, Cheng Zhu, Zaili Lin, Jianmai Shi, Weiming Zhang

**Affiliations:** 1 Science and Technology on Information Systems Engineering Laboratory, National University of Defense Technology, Changsha, Hunan, China; 2 College of Mechanical and Power Engineering, Chongqing University of Science and Technology, Chongqing, China; Southwest University, CHINA

## Abstract

This paper studies a reliable facility location problem with facility protection that aims to hedge against random facility disruptions by both strategically protecting some facilities and using backup facilities for the demands. An Integer Programming model is proposed for this problem, in which the failure probabilities of facilities are site-specific. A solution approach combining Lagrangian Relaxation and local search is proposed and is demonstrated to be both effective and efficient based on computational experiments on random numerical examples with 49, 88, 150 and 263 nodes in the network. A real case study for a 100-city network in Hunan province, China, is presented, based on which the properties of the model are discussed and some managerial insights are analyzed.

## Introduction

Facilities are critical infrastructures of service networks and supply networks, and locating facilities properly is highly important for providing products, information or services to customers both efficiently and sustainably. In real life, facilities may occasionally fail to work due to disruptions such as earthquakes, hurricanes, terrorist attacks, and equipment breakdowns. These disruptions substantially increase both the service costs and customer dissatisfaction because customers may have to seek service from locations other than their preferred facilities or because their demand may be delayed or even abandoned after a disruption, which may lead to higher transportation costs, order delays, or a loss of market shares.

Unfortunately, some disruptions may compromise the performance of the whole supply network and result in devastating consequences. For example, in 2001, an eight-minute fire halted a semiconductor factory belonging to Philips in New Mexico, USA, for 9 months, which indirectly caused one of its customers, Ericsson, to lose USD 2.34 billion [1].In 2011, a 8.9-magnitude earthquake and its resulting tsunami struck Japan, which severely affected component plants of several industries; the production lines of many international companies were shut down for long periods due to part shortages from their Japanese suppliers [2, 3].

Facility location decisions are strategic: once a facility network is constructed, it is costly and time-consuming to reconfigure and rebuild. Additionally, recourses are always limited, and restoration processes can be very lengthy after a disputation. These factors highlight the need for taking facility disruptions into account and designing a robust facility network that has the abilities to hedge against disruptions.

This paper studies a reliable facility location problem with facility protection, henceforth abbreviated RFLPFP, that aims to increase the reliability of a facility network by both protecting some facilities and assigning customers to backup facilities. More specifically, RFLPFP assumes that facilities may fail independently and with site-specific failure probabilities but that their reliability can be protected through extra investments. In RFLPFP, each customer should be assigned to a primary facility, which can be either a reliable facility or an unreliable facility. If the primary facility of a customer is unreliable, she should be assigned to a protected reliable facility as well, which works as her backup facility. Therefore, the customer can obtain emergency services from her backup facility when her primary facility fails. This protection and backup mechanism ensures that all customers are served, even if some facilities fail. The objective of RFLPFP is to determine the facility location and protection decisions as well as customer assignments to minimize the fixed charges and expected service cost.

This work contributes to the current literature in three main aspects. First, the facility location and protection problem is formulated as an integer programming model that considers the site-specific failure probabilities. Compared to the literature on reliable facility location problems with facility protection, this new integer programming model more precisely captures the impact of different failure probabilities when facilities are located at different places. Second, a solution approach is developed by combining Lagrangian relaxation with some local search strategies. The performance of the approach is compared with the commercial optimization solver CPLEX by using random numerical examples with different sizes. Third, a practical case study is presented, based on which the impact of facility disruption probabilities, protection cost and emergency supply cost on the location decisions is analyzed.

The rest of this paper is organized as follows: in Section 2, some relevant studies are reviewed. In Section 3, the RFLPFP problem is described, and a new linear integer programming model is proposed. In Section 4, based on the formulation, a solution method combining Lagrangian relaxation and local search is presented. In Section 5, some computational experiments are conducted on some benchmark datasets, and comparisons of the developed algorithm and the CPLEX are provided. Additionally, a sensitivity analysis is conducted on a case example, and some managerial insights are presented. Finally, in Section 6, we provide a conclusion and suggest several future research directions.

## Literature review

The facility location problem is both a classical optimization problem and a fundamental problem in designing supply or service networks, and it has been extensively studied over the past several decades. There is an abundant body of literature on both deterministic and stochastic facility location problems [4–8].However, a majority of the literature addresses primarily demand and cost uncertainties [8],and relatively fewer studies consider the influence of facility disruptions. Most recently, both academics and practitioners have realized that facility disruptions may be triggered by various factors and may occur frequently, and an increasing number of studies are investigating ways to improve the reliability of facility networks by planning for facility disruptions [9–11].

There are two main research streams on tackling facility disruptions. The first one seeks to improve the availability of facilities by increasing redundancy and utilizing backups. These models always explicitly consider the disruption probabilities when designing a facility network. Snyder et al. [12] study the reliable P-median problem and the reliable uncapacitated fixed-charge location problem, which simultaneously optimize the operating cost under regular circumstances and the expected cost when disruption occurs. By analyzing the trade-off curves of both costs, the authors note that substantial improvements in reliability can always be obtained with only slight increases in the regular cost. Cui et al. [13] extend Snyder’s work by relaxing the assumption of uniform disruption probability and allow site-specific disruption probabilities; they thus design a Lagrangian relaxation-based algorithm and a continuous approximation algorithm to solve the problem. Berman et al. [14] study the reliable P-median problem on a network and propose several exact and heuristic algorithms, ultimately revealing that facilities become more centralized or even co-located as the failure probability increases. Shen et al. [15] propose a scenario-based stochastic program and a nonlinear integer program for the reliable facility location problem with heterogeneous failure probabilities. They prove that both models are equivalent and propose a constant-ratio approximation algorithm for the uniform case. Li et al. [16] consider the correlated effect of disruptions and propose a continuous approximation approach for the reliable facility location problem.

A common feature of the above papers is that they all utilize the multiple-level assignment strategy to increase the reliability of a facility network; that is, each customer should be assigned to a group of facilities that are ordered by levels. Once a customer’s *n*th-level facility is disrupted, she will seek service from her (*n* + 1)th-level operational facility, and so on. Ultimately, a customer is served by an operational facility, or her demand is abandoned if all of her assigned facilities fail; in the latter case, a penalty is charged. Though this multi-level backup mechanism can improve the availability of facilities, it also increases the operational and managerial complexities because each customer must set up connections with multiple facilities and vice versa. A more complicated situation emerges when prior information on the operational states of facilities is unknown to a customer before reaching it; therefore, the customer may have to visit several disrupted facilities before finding an operational one [17–19].

The second stream of related work focuses on improving facility availability by explicitly protecting or fortifying some of the most critical facilities. In reality, various protection measures are available, such as installing structural reinforcements, adding built-in redundancies, improving monitoring and security guarding, buying insurance and using outsourcing. Most of the papers considering facility protection assume a context of deliberate attacks, i.e., where an intelligent adversary intentionally tries to interdict the facility network to maximize the losses and where, in contrast, a defender protects some of the most critical components to mitigate the effect of the attacks.

Scaparra et al. [20] study the r-interdiction median problem with fortification, which selects *q* facilities to protect among the total *p* existing facilities such that the impact of the most disruptive attack on *r*(*q* + *r* < *p*) unprotected facilities is minimized. Aksen et al. [21] study the protection resource-constrained facility protection problems, in which the number of protected facilities is not predetermined but should be computed optimally while satisfying the protection resource constraints. Zhu et al. [22] further consider the probabilistic protection problem, which assumes that the probability of being interdicted for a facility decreases as more protection resources are allocated to it. Liberatore et al. [23] consider the correlation between the facilities, use a two-dimensional correlation matrix to model the interdependence between facilities and present a location-attack-assignment tri-level model. A location-hardening problem with the objective of minimizing the maximum distance from a customer to its closest operational facility after facility disruptions is studied in [24]. Almost all of these protection models focus on the worst case, and no failure probability information about the facilities is considered.

The objective of this paper is to combine the use of backup mechanisms with facility protection to hedge against random facility disruptions. A few studies investigating such types of reliable facility location problems are those by Lim et al. [25] and Li et al. [26, 27]. Lim et al. [25] study a reliable facility location problem in which facility fortification options and single-level backup strategies are adopted to improve the availability of the facilities. A linear integer programming model is presented to formulate the problem, and the efficiency of the model is illustrated through examples in which all of the facilities have the same failure probability; however, there is a lack of deep analysis for solving a problem with site-specific probabilities. Li et al. [26, 27] extend the work of Lim et al. by further considering the protection budget constraint, and they make the strong assumption that a backup facility is always available even when it is not protected.

Different from Lim’s model, this paper proposes a more general linear integer programming model that can address site-specific failure probabilities; therefore, it is more suitable for the random disruptions that are triggered by natural or accidental events. Additionally, different from Li’s models, in our model, only a protected reliable facility can work as a backup facility, and a customer must be backed up to a protected facility if her primary facility is unreliable. This mechanism ensures that customers are always served. Compared to the multi-level assignment mechanisms presented in the existing literature, this paper adopts a single-level backup mechanism to hedge against facility disruptions, as performed in [25–27], which makes the facility-customer relationship simpler and clearer, thereby reducing the operational complexity of the system. Furthermore, the time latency for emergency services can be reduced because a backup facility is always available, which is critical, especially when the customers’ needs are time-sensitive.

## Mathematical formulation

RFLPFP is an extension of the classical facility location problem (FLP). It is assumed that facilities may fail independently and with site-specific failure probabilities, and the probability information can be estimated by analyzing the historical data. We also assume that the reliability of facilities can be protected through extra investments. Possible protection measures include built-in redundancies, structural reinforcements, preventive monitoring and safety guarding, and outsourcing. The protected facilities never fail, but they are more expensive than their unprotected counterparts are. Therefore, there are two types of facilities in the system: the unreliable regular ones and the reliable protected ones. Each customer should be assigned to a primary facility, which can be either a regular facility or a protected facility. If the primary facility of a customer is a regular one, she should be assigned to a protected facility as well, which works as her backup facility. In this way, the customer can obtain emergency services from her backup facility when her primary facility fails. This protection and backup mechanism ensures that each customer is served with a probability of 1. RFLPFP aims to determine the facility location and protection decisions as well as customer assignments to minimize the fixed charges and expected service cost.

To formulate the problem as an integer programming model, we use the following notations:

**Notations**
*I*: set of customer points, indexed by *i*
*J*: set of potential facility sites, indexed by *j*
*h*_*i*_: demand of customer *i*
*q*_*j*_: failure probability of the unreliable facility opened at site *j*
$f_{j}^{U}$: fixed charge of opening an unreliable facility at site *j*
$f_{j}^{R}$: fixed charge of opening a reliable facility at site *j*, and $f_{j}^{R}>f_{j}^{U}$
$d_{ij}^{P}$: unit service cost when customer *i* is served by her primary facility opened at site *j*.
$d_{ij}^{B}$: unit service cost when customer *i* is served by her backup facility opened at site *j*. We assume that $d_{ij}^{B}\geq d_{ij}^{P}$ to reflect that the emergency cost when customer *i* is served by her backup facility is not lower than the regular cost when customer *i* is served by her primary facility.

**Decision variables**
$X_{j}^{R}=1$ if a reliable facility is opened at site *j*; $X_{j}^{R}=0$ otherwise.
$X_{j}^{U}=1$ if an unreliable facility is opened at site *j*; $X_{j}^{U}=1$ otherwise.
*Y*_*ikj*_ = 1 if customer *i* is assigned to an unreliable facility *k* as her primary facility and to a reliable facility *j* as her backup facility; *Y*_*ikj*_ = 0 otherwise.
*Z*_*ij*_ = 1 if customer *i* is assigned to a reliable facility *j* as her primary facility; *Z*_*ij*_ = 0 otherwise.

The linear integer programming model for the RFLPFP is as follows.

$$\left(P1\right)\;\min\sum_{\forall j\in J}f_{j}^{U}X_{j}^{U}+\sum_{\forall j\in J}f_{j}^{R}X_{j}^{R}+\sum_{\forall i\in I}\sum_{\forall j\in J}h_{i}d_{ij}^{P}Z_{ij}+\sum_{\forall i\in I}\sum_{\forall k\in J,k\neq j}\sum_{\forall j\in J}\left(h_{i}d_{ik}^{P}\left(1-q_{k}\right)Y_{ikj}+h_{i}d_{ij}^{B}q_{k}Y_{ikj}\right)$$ (1)

$$\begin{array}{l} s.t.\\ \;X_{j}^{U}+X_{j}^{R}\leq 1,\forall j\in J \end{array}$$ (2)

$$\sum_{\forall k\in J,k\neq j}\sum_{\forall j\in J}Y_{ikj}+\sum_{\forall j\in J}Z_{ij}=1,\forall i\in I$$ (3)

$$Z_{ij}\leq X_{j}^{R},\forall i\in I,\forall j\in J$$ (4)

$$\sum_{\forall k\in J,k\neq j}Y_{ijk}\leq X_{j}^{U},\forall i\in I,\forall j\in J$$ (5)

$$\sum_{\forall k\in J,k\neq j}Y_{ikj}\leq X_{j}^{R},\forall i\in I,\forall j\in J$$ (6)

$$\sum_{\forall j\in J}X_{j}^{R}\geq 1$$ (7)

$$X_{j}^{R},X_{j}^{U}\in \left\{0,1\right\},\forall j\in J$$ (8)

$$Z_{ij}\in \left\{0,1\right\},\forall i\in I,\forall j\in J$$ (9)

$$Y_{ikj}\in \left\{0,1\right\},\forall i\in I,\forall j,k\neq j\in J$$ (10)

The objective function Eq 1 is to minimize the overall costs, including the fixed cost of opened unreliable and reliable facilities, the deterministic service cost for customers who are served by a reliable facility, and the expected service cost for customers who are served by a primary facility and a backup facility. Constraint 2 denote that either a reliable facility or an unreliable facility, but not both, can be opened at a single site. Constraint 3 state that a customer is assigned either directly to a reliable primary facility or to an unreliable facility as her primary facility and a protected facility as her backup facility. Constraint 4 state that if a customer is assigned to only one layer facility, then that facility should be reliable. Constraints 5 and 6 indicate that if a customer is assigned to two layer facilities, then the primary facility should be unreliable and the backup facility should be reliable. Constraint 7 states that at least one reliable facility should be opened, which is a redundant constraint and can be derived by combining constraints 3, 4 and 6, but we use it to tighten the bound when we develop the Lagrangian relaxation algorithm. Constraints 8–10 are the integrality constraints.

## Solution approach based on Lagrangian Relaxation

When all of the facilities have a failure probability of 0, the RFLPFP is reduced to the classical uncapacitated fixed-charge facility location problem, which is NP hard. Hence, RFLPFP is also NP hard. The model contains 2|*J*| + |*I*| × |*J*| × |*J*| binary variables and |*J*| + |*I*| + 3|*I*| × |*J*| + 1 constraints. In this section, we present a Lagrangian relaxation-based approach to solve the problem. The main ideas of the Lagrangian relaxation approach are the following: first, the “hard” constraints of the original problem are relaxed, which leads to a relaxation problem that can be solved relatively easily and helps in obtaining a lower bound for the original problem. Then, based on the solution to the relaxation problem, we can construct a feasible solution to the original problem, which provides an upper bound. Usually, some search algorithms can be used to improve the upper bound solution. The Lagrange multipliers are then adjusted to reduce the amount of constraint violation, and we again obtain a new relaxation problem that corresponds to the updated Lagrange multipliers. This procedure repeats until some stopping conditions are met. The details of the algorithm are explained in the following subsections.

We relax constraint 3 with the Lagrange multipliers *λ*, relax constraint 5 with Lagrange multipliers *μ* and obtain the corresponding Lagrangian relaxation problem.

$$\begin{array}{l} Z_{LR}(\lambda ,\mu \geq 0)=\underset{X^{R},X^{U},Z,Y}{\text{min}}\sum_{\forall j\in J}f_{j}^{U}X_{j}^{U}+\sum_{\forall j\in J}f_{j}^{R}X_{j}^{R}+\sum_{\forall i\in I}\sum_{\forall j\in J}h_{i}d_{ij}^{P}Z_{ij}+\sum_{\forall i\in I}\sum_{\forall k\in J,k\neq j}\sum_{\forall j\in J}h_{i}(d_{ik}^{P}(1-q_{k})+d_{ij}^{B}q_{k})Y_{ikj}+\sum_{\forall i\in I}\lambda_{i}(1-\sum_{\forall k\in J,k\neq j}\sum_{\forall j\in J}Y_{ikj}-\sum_{\forall j\in J}Z_{ij})+\sum_{\forall i\in I}\sum_{\forall j\in J}\mu_{ij}(\sum_{\forall k\in J,k\neq j}Y_{ijk}-X_{j}^{U})\\ s.t.2,\;4,\;6,\;7,\;8,\;9,\;10 \end{array}$$ (11)

Because ∑_∀*i* ∈ *I*_∑_∀*j* ∈ *J*_∑_∀*k* ∈ *J*,*k* ≠ *j*_ *μ*_*ij*_ *Y*_*ijk*_ = ∑_∀*i* ∈ *I*_∑_∀*k* ∈ *J*_∑_∀*j* ∈ *J*,*j* ≠ *k*_ *μ*_*ik*_ *Y*_*ikj*_ = ∑_∀*i* ∈ *I*_∑_∀*k* ∈ *J*,*k* ≠ *j*_∑_∀*j* ∈ *J*_ *μ*_*ik*_ *Y*_*ikj*_, the above formulation can be rewritten as

$$\begin{array}{l} Z_{LR}(\lambda ,\mu \geq 0)=\underset{X^{R},X^{U},Z,Y}{\text{min}}\sum_{\forall j\in J}f_{j}^{R}X_{j}^{R}+\sum_{\forall j\in J}\left(f_{j}^{U}-\sum_{\forall i\in I}\mu_{ij}\right)X_{j}^{U}+\sum_{\forall i\in I}\sum_{\forall j\in J}\left(h_{i}d_{ij}^{P}-\lambda_{i}\right)Z_{ij}+\sum_{\forall i\in I}\sum_{\forall k\in J,k\neq j}\sum_{\forall j\in J}(h_{i}d_{ik}^{P}(1-q_{k})+h_{i}d_{ij}^{B}q_{k}+\mu_{ik}-\lambda_{i})Y_{ikj}+\sum_{\forall i\in I}\lambda_{i}\\ s.t.2,\;4,\;6,\;7,\;8,\;9,\;10 \end{array}$$ (12)

For the sake of brevity, we rewrite the above formulation 12 as follows:

$$\begin{array}{l} Z_{LR}(\lambda ,\mu \geq 0)=\underset{X^{R},X^{U},Z,Y}{\text{min}}\sum_{\forall j\in J}f_{j}^{R}X_{j}^{R}+\sum_{\forall j\in J}\alpha_{j}X_{j}^{U}+\sum_{\forall i\in I}\sum_{\forall j\in J}\beta_{ij}Z_{ij}+\sum_{\forall i\in I}\sum_{\forall k\in J,k\neq j}\sum_{\forall j\in J}\gamma_{ikj}Y_{ikj}+\sum_{\forall i\in I}\lambda_{i}\\ s.t.2,\;4,\;6,\;7,\;8,\;9,\;10 \end{array}$$ (13)

where $\alpha_{j}=f_{j}^{U}-\sum_{\forall i\in I}\mu_{ij}$, $\beta_{ij}=h_{i}d_{ij}^{P}-\lambda_{i}$, and $\gamma_{ikj}=h_{i}d_{ik}^{P}\left(1-q_{k}\right)+h_{i}d_{ij}^{B}q_{k}+\mu_{ik}-\lambda_{i}$.

For the given Lagrange multipliers *λ* and *μ*, Eq 13 provides a lower bound for the original problem 1. The Lagrangian dual problem involves finding the maximal lower bound of the original problem 1 by calculating the optimal Lagrange multipliers *λ* and *μ*, which can be expressed as follows:

$$\begin{array}{l} LD(\lambda ,\mu \geq 0)=\underset{\lambda ,\mu \geq 0}{\text{max}}\underset{X^{R},X^{U},Z,Y}{\text{min}}\sum_{\forall j\in J}f_{j}^{R}X_{j}^{R}+\sum_{\forall j\in J}\alpha_{j}X_{j}^{U}+\sum_{\forall i\in I}\sum_{\forall j\in J}\beta_{ij}Z_{ij}+\sum_{\forall i\in I}\sum_{\forall k\in J,k\neq j}\sum_{\forall j\in J}\gamma_{ikj}Y_{ikj}+\sum_{\forall i\in I}\lambda_{i}\\ s.t.2,\;4,\;6,\;7,\;8,\;9,\;10 \end{array}$$ (14)

The dual problem 14 can be solved by using the sub-gradient algorithm [28, 29].

### Solving the Lagrangian relaxation problem

Solving the relaxation problem, that is, solving the minimization problem 13 for the given Lagrange multipliers *λ* and *μ*, is a very critical step.

If we ignore constraint 7, which stipulates the restriction that at least one reliable facility is opened, the Lagrangian relaxation problem *Z*_*LR*_(*λ*, *μ* ≥ 0) is separable on *j*, and it can be decomposed into |*J*| independent subproblems, which can be solved relatively easily. Specifically, the subproblem corresponding to site *j* while dropping Eq 7 can be expressed as shown below:

$$\left(P_{j}\right)\;\min f_{j}^{R}X_{j}^{R}+\alpha_{j}X_{j}^{U}+\sum_{\forall i\in I}\beta_{ij}Z_{ij}+\sum_{\forall i\in I}\sum_{\forall k\in J,k\neq j}\gamma_{ikj}Y_{ikj}$$ (15)

$$\begin{array}{l} s.t.\\ \;X_{j}^{U}+X_{j}^{R}\leq 1 \end{array}$$ (16)

$$Z_{ij}\leq X_{j}^{R},\forall i\in I$$ (17)

$$\sum_{\forall k\in J,k\neq j}Y_{ikj}\leq X_{j}^{R},\forall i\in I$$ (18)

$$X_{j}^{R},X_{j}^{U}\in \left\{0,1\right\}$$ (19)

$$Z_{ij}\in \left\{0,1\right\},\forall i\in I$$ (20)

$$Y_{ikj}\in \left\{0,1\right\},\forall i\in I,\forall k\neq j\in J$$ (21)

For each site *j*, there are three possible states, i.e., an unreliable facility is opened, a reliable facility is opened, or no facility is opened. We first compute the objective value Eq 15 corresponding to each state; then, the state with the minimum objective value will be selected, and the values for the decision variables $\left(X_{j}^{U},X_{j}^{R},Z_{ij},Y_{ikj}\right)$ will be set according to the selected state. The details for these three cases are presented below.

Case 1: Assume that $X_{j}^{U}=1$, which means that one unreliable facility is opened at *j*. We denote the objective value for this case as $V_{j}^{U}$. Because when $X_{j}^{U}=1$, we have $X_{j}^{R}=0,Z_{ij}=0,\forall i\in I,Y_{ikj}=0,\forall i\in I,\forall k\neq j\in J$, it can be easily seen from expression 15 that $V_{j}^{U}=\alpha_{j}$.

Case 2: Assume that $X_{j}^{R}=1$, which means that one reliable facility is built at *j*. This case is more complicated than case 1. We denote the objective value for this case as $V_{j}^{R}$. When $X_{j}^{R}=1$, according to constraints 16–18, we can derive that $X_{j}^{U}=0,Z_{ij}\leq 1,\forall i\in I$, and ∑_∀*k* ∈ *J*,*k* ≠ *j*_ *Y*_*ikj*_ ≤ 1, ∀*i* ∈ *I*. We then obtain $V_{j}^{R}=f_{j}^{R}+\sum_{\forall i\in I}\min\left(\beta_{ij},0\right)+\sum_{\forall i\in I}\min\left(\min_{\forall k\in J,k\neq j}\gamma_{ikj},0\right)$, and the values for *Z*_*ij*_ and *Y*_*ikj*_ are set as follows:

$$Z_{ij}=\left\{\begin{array}{cc} 1, & \text{if}\;\beta_{ij}<0;\\ 0, & \text{otherwise}. \end{array}\right.,\forall i\in I.$$ (22)

$$Y_{ikj}=\left\{\begin{array}{cc} 1, & \text{if}\;\gamma_{ikj}<0\;\text{and}\;k={\text{argmin}}_{\forall k\in J,k\neq j}\gamma_{ikj};\\ 0, & \text{otherwise}. \end{array}\right.,\forall i\in I,\forall k\in J,k\neq j.$$ (23)

Case 3: Assume that $X_{j}^{U}=X_{j}^{R}=0$, which means that no facility is built on site *j*. In this case, we can easily derive that *Z*_*ij*_ = 0, ∀*i* ∈ *I*,*Y*_*ikj*_ = 0, ∀*i* ∈ *I*, ∀*k* ≠ *j* ∈ *J*. Therefore, its contribution to the objective function is 0.

Next, still ignoring constraint 7, we determine the state for every site *j* as follows: If $\min\left(V_{j}^{U},V_{j}^{R},0\right)=V_{j}^{U}<0$, we set $X_{j}^{U}=1$, and the values for variables $\left(X_{j}^{R},Z_{ij},Y_{ikj}\right)$ associated with *j* are set as stated for case 1. If $\min\left(V_{j}^{U},V_{j}^{R},0\right)=V_{j}^{R}<0$, we set $X_{j}^{R}=1$, and the values for $\left(X_{j}^{U},Z_{ij},Y_{ikj}\right)$ are set as stated for case 2. If $\min(V_{j}^{U},V_{j}^{R},0)=0$, we set all of the variables $\left(X_{j}^{R},X_{j}^{U},Z_{ij},Y_{ikj}\right)$ as 0.

We now consider constraint 7. Let $J^{R}=\left\{j|X_{j}^{R}=1,j\in J\right\}$ denote the set of sites at which a reliable facility is opened, let $J^{U}=\left\{j|X_{j}^{U}=1,j\in J\right\}$ denote the set of sites at which an unreliable facility is opened, and let *J*^*C*^ = *J*∖{*J*^*U*^,*J*^*R*^} denote the rest of the sites at which no facility has been opened yet. If we have at least one reliable facility opened, i.e., |*J*^*R*^| ≥ 1, then the lower bound has been achieved because the constraint 7 has been satisfied already. Otherwise, we need to open a reliable facility at a site in either *J*^*U*^ or *J*^*C*^. We determine the best location for opening a reliable facility as follows:

Let $VJ_{j}^{U}=V_{j}^{R}-V_{j}^{U},j\in J^{U}$ and $VJ_{j}^{C}=V_{j}^{R},j\in J^{C}$. If $\min_{j\in J^{U}}VJ_{j}^{U}<\min_{j\in J^{C}}VJ_{j}^{C}$, we set $X_{j^{*}}^{U}=1$, where $j^{*}=argmin_{j\in J^{U}}VJ_{j}^{U}$; otherwise, we set $X_{j^{*}}^{R}=1$, where $j^{*}=argmin_{j\in J^{C}}VJ_{j}^{C}$.

Once the location j* for opening the reliable facility is determined, we set the values for the other decision variables $\left(X_{j^{*}}^{U},Z_{ij^{*}},Y_{ikj^{*}}\right)$ associated with j* according to case 2 as specified above.

By solving the Lagrangian relaxation problem, we obtain the lower bound:

$$Z_{LR}\left(\lambda ,\mu \geq 0\right)=\sum_{\forall j\in J}\left(V_{j}^{U}X_{j}^{U}+V_{j}^{R}X_{j}^{R}\right)+\sum_{\forall i\in I}\lambda_{i}.$$ (24)

### Computation of the upper bound

After solving the Lagrangian relaxation problem, we obtain a feasible location configuration $\left(X_{j}^{U},X_{j}^{R}\right)$, which may not be the optimal location configuration for the original problem 1 but can be used to construct a feasible solution that provides an upper bound for Eq 1. We let the location decisions of the feasible solution be the same as that of the relaxation problem; thus, the customer assignment of the feasible solution is the optimal assignment corresponding to the location configuration, which is computed as described below.

Let *J*^*R*^ be the set of opened reliable facilities, and let *J*^*U*^ be the set of opened unreliable facilities. First, we provide some properties:

**Property 1:** If customer *i* has a reliable facility j* as her backup facility, then $j^{*}=argmin_{j\in J^{R}}d_{ij}^{B}$.

Proof: Assume that *i* has an unreliable facility *k* ∈ *J*^*U*^ as her primary facility and another reliable facility *j*^∘^ ∈ *J*^*R*^,*j*^∘^ ≠ j* as her backup facility; then, the expected service cost for *i* is $h_{i}d_{ik}^{P}\left(1-q_{k}\right)+h_{i}d_{ij^{\circ }}^{B}q_{k}$. As $j^{*}=argmin_{j\in J^{R}}d_{ij}^{B}$, we have $h_{i}d_{ik}^{P}\left(1-q_{k}\right)+h_{i}d_{ij^{\circ }}^{B}q_{k}\geq h_{i}d_{ik}^{P}\left(1-q_{k}\right)+h_{i}d_{ij^{*}}^{B}q_{k}$; hence, it is better to have j* as her backup facility.

**Property 2:** Let $j^{*}=argmin_{j\in J^{R}}d_{ij}^{B}$; if customer *i* has an unreliable facility k* as her primary facility, then $k^{*}=argmin_{k\in J^{U}}\left(d_{ik}^{P}\left(1-q_{k}\right)+d_{ij^{*}}^{B}q_{k}\right)$.

Proof: Assume *i* has another unreliable facility *l* ∈ *J*^*U*^,*l* ≠ *k* as her primary facility and that the expected service cost for *i* is $h_{i}d_{il}^{P}\left(1-q_{l}\right)+h_{i}d_{ij^{*}}^{B}q_{l}$. As $k^{*}=argmin_{k\in J^{U}}\left(d_{ik}^{P}\left(1-q_{k}\right)+d_{ij^{*}}^{B}q_{k}\right)$, we have $h_{i}d_{il}^{P}\left(1-q_{l}\right)+h_{i}d_{ij^{*}}^{B}q_{l}\geq h_{i}d_{ik^{*}}^{P}\left(1-q_{k^{*}}\right)+h_{i}d_{ij^{*}}^{B}q_{k^{*}}$; hence, it is better to have k* as her primary facility.

**Property 3:** Let $j^{*}=argmin_{j\in J^{R}}d_{ij}^{B}$, $k^{*}=argmin_{k\in J^{U}}\left(d_{ik}^{P}\left(1-q_{k}\right)+d_{ij^{*}}^{B}q_{k}\right)$, and $j^{\circ }=argmin_{j\in J^{R}}d_{ij}^{P}$. Then, if $d_{ij^{\circ }}^{P}\leq \left(d_{ik^{*}}^{P}\left(1-q_{k^{*}}\right)+d_{ij^{*}}^{B}q_{k^{*}}\right)$, customer *i* should be assigned to *j*^∘^ directly; otherwise, she should be assigned to k* as her primary facility and j* as her backup facility.

Proof: Customer *i* is assigned either directly to a reliable facility or to an unreliable facility as her primary facility and a reliable facility as her backup facility. For the former case, the service cost is *h*_*i*_ *d*_*ij*^∘^_; for the latter case, the service cost is $h_{i}\left(d_{ik^{*}}^{P}\left(1-q_{k^{*}}\right)+d_{ij^{*}}^{B}q_{k^{*}}\right)$. Obviously, the assignment with the smaller service cost will be selected.

Based on the above properties, the procedure for determining the optimal assignment for customer *i* is straightforward and can be performed in *O*(|*J*|) as follows:

First, we compute $j^{\circ }=argmin_{j\in J^{R}}d_{ij}^{P}$, $j^{*}=argmin_{j\in J^{R}}d_{ij}^{B}$, and $k^{*}=argmin_{k\in J^{U}}\left(d_{ik}^{P}\left(1-q_{k}\right)+d_{ij^{*}}^{B}q_{k}\right)$.

Then, if $d_{ij^{\circ }}^{P}\leq d_{ik^{*}}^{P}\left(1-q_{k^{*}}\right)+d_{ij^{*}}^{B}q_{k^{*}}$, we set

$$Z_{ij}=\left\{\begin{array}{cc} 1, & \text{if}\;j=j^{\circ };\\ 0, & \text{otherwise}. \end{array}\right.,\text{and}\;Y_{ikj}=0,\forall k,\forall j\in J.$$ (25)

Otherwise, we set

$$Y_{ikj}=\left\{\begin{array}{cc} 1, & \text{if}\;k=k^{*}\;\text{and}\;j=j^{*};\\ 0, & \text{otherwise}. \end{array}\right.,\text{and}\;Z_{ij}=0,\forall j\in J.$$ (26)

The objective value corresponding to this feasible solution is

$$\sum_{\forall j\in J}\left(f_{j}^{U}X_{j}^{U}+f_{j}^{R}X_{j}^{R}\right)+\sum_{\forall i\in I}\min\left(h_{i}d_{ij^{\circ }}^{P},h_{i}d_{ik^{*}}^{P}\left(1-q_{k^{*}}\right)+h_{i}d_{ij^{*}}^{B}q_{k^{*}}\right),$$ (24)

which is an upper bound for the original problem.

### Local search to improve the upper bound solution

As is commonly used in Lagrangian relaxation-based algorithms, a local search procedure is designed to improve the upper bound solution, which is demonstrated to be both effective and efficient based on the computational experiments.

**Fig 1:**
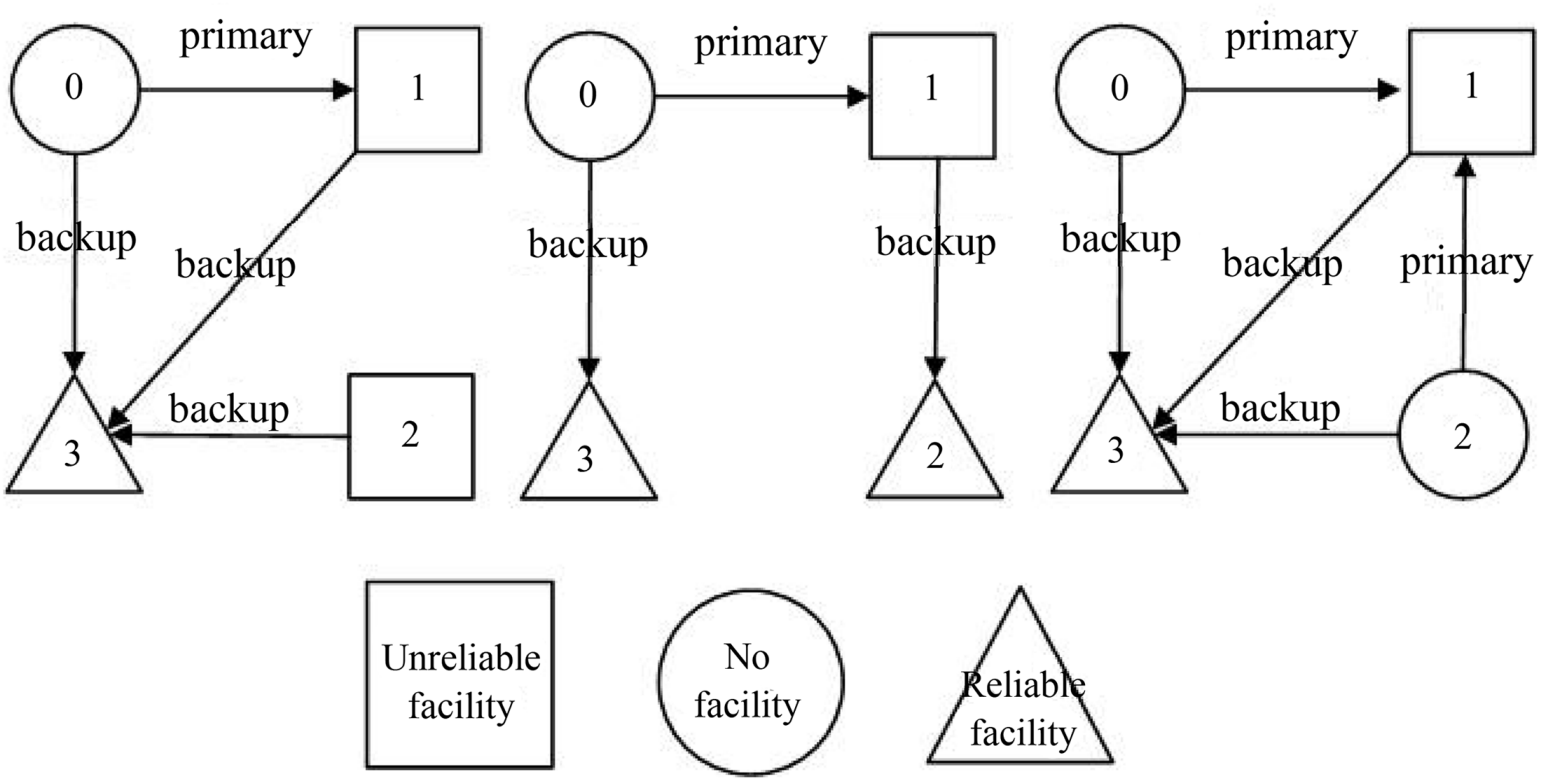
Illustrative example for the local moves.

The local search is based on the switching states of the sites. Specifically, each site has only three possible states as stated above: no facility opened, denoted as 0; one unreliable facility opened, denoted as 1; or one reliable facility opened, denoted as 2. Hence, for each site, there are two possible moves, i.e., switching from the current state to one of the two other states. It should be noted that when a site that has a reliable facility opened switches its state, i.e., 2→0 or 2→1, it should be guaranteed that at least one reliable facility remains opened in the system. For each move, the customer assignment and objective value corresponding to the new location configuration can be computed by using the method stated above. An example of the local moves is depicted in Fig 1, where the left graph shows the initial location and assignment configurations, the middle one shows the configurations when site 2 moves from state 1 to state 2, and the right graph shows the configurations when site 2 moves from state 1 to state 0.

We use a steepest descent strategy to choose the moves. For each inner loop, the move with the highest cost savings will be selected, and this process will be repeated until no cost savings can be obtained from any moves of any site. The pseudocode for the local search procedure is presented as “Algorithm 1”:

**Algorithm 1** The local search procedure

*upperBound* ← objective value of the upper bound solution

**while** true **do**

 **for all** *j* ∈ *J* **do**

  **if** *j* is the only site with a reliable facility opened **then**

   continue;

  **else**

   compute the objective value corresponding to move1 and move2 for *j*, denoted as *costMove*1*J* and *costMove*2*J*, respectively;

   *upperBound* ← *min*(*upperBound*, *costMove*1*J*, *costMove*2*J*);

  **end if**

 **end for**

 **if** *upperBound* is updated **then**

  select the move that leads to *upperBound*, i.e., update the location decisions and assignment decisions according to that move;

 **else**

  break;

 **end if**

**end while**

Because each site has at most two possible moves and computing the objective value for each move requires *O*(|*I*||*J*|) effort, the time complexity of the internal loop of “Algorithm 1” is *O*(|*I*||*J*|^2^). To reduce the overall computational time, we only run the local search when the minimal upper bound obtained thus far is updated. This strategy is shown to be very efficient in the computational experiments.

### Sub-gradient algorithm

As stated above, the Lagrangian dual problem

$$\begin{array}{c} \left(LD)\;\underset{\lambda ,\mu \geq 0}{\text{max}}\;Z_{LR}(\lambda ,\;\mu \right)\\ s.t.2,\;4,\;6,\;7,\;8,\;9,\;10 \end{array}$$ (28)

can be solved using the sub-gradient algorithm, which improves the lower bound by iteratively adjusting the Lagrange multipliers *λ* and *μ*. During this process, the upper bound solution can be also improved. This process repeats until some stopping criteria are met. The whole procedure for the sub-gradient algorithm is explained below:

Step 1: Generate an initial upper bound solution and initialize some parameters.

Because a location configuration with at least one reliable facility opened automatically leads to a feasible solution, we greedily select the site $j^{*}=argmin_{j\in J}\sum_{i\in I}h_{i}d_{ij}^{P}$ to open a reliable facility and assign all customers to j* directly. Then, we use the local search procedure to improve this solution and obtain an initial upper bound *UB*. *UB* is also used to denote the best upper bound found thus far. We initialize the iteration counter *iterNum* = 0, the Lagrange multipliers $\lambda_{i}^{\left(0\right)}=0,\forall i\in I$, $\mu_{ij}^{\left(0\right)}=0,\forall i\in I,\forall j\in J$, and the direction vectors $d_{i}^{\lambda (0)}=d_{ij}^{\mu (0)}=0,\forall i\in I,\forall j\in J$.

Step 2: Compute the lower bound solution and update the upper bound solution at the *n*th iteration.

Specifically, we can obtain a lower bound solution (*X*^*U*^, *X*^*R*^, *Y*, *Z*) by solving the Lagrangian relaxation problem *Z*_*LR*_(*λ*^(*n*)^, *μ*^(*n*)^) at the *n*th iteration, denoting its value as *LB*^(*n*)^. Based on the lower bound solution, we construct a feasible solution as stated above. If the value of the feasible solution is smaller than *UB*, we use the local search procedure to improve it and update *UB* accordingly.

Step 3: Update the Lagrange multipliers.

First, we compute the move directions for the Lagrange multipliers *λ* and *μ* at the *n*th iteration, denoted as $d_{i}^{\lambda (n)}$ and $d_{ij}^{\mu (n)}$, respectively. They are computed as shown below:

$$d_{i}^{\lambda (n)}=\left(1-\sum_{\forall k\in J,k\neq j}\sum_{\forall j\in J}Y_{ikj}-\sum_{\forall j\in J}Z_{ij}\right)+C\cdot d_{i}^{\lambda (n-1)}$$ (29)

$$d_{ij}^{\mu (n)}=\left(\sum_{\forall k\in J,k\neq j}Y_{ijk}-X_{j}^{U}\right)+C\cdot d_{ij}^{\mu (n-1)}$$ (30)

where *C* is a Crowder damping constant [30].We set *C* = 0.3 to the same value used in [25]. Then, we compute the step size *t*^(*n*)^ for the Lagrange multipliers as shown below:

$$t^{\left(n\right)}=\theta^{\left(n\right)}\frac{UB-LB^{\left(n\right)}}{\sum_{\forall i\in I}{\left(d_{i}^{\lambda (n)}\right)}^{2}+\sum_{\forall i\in I}\sum_{\forall j\in J}{\left(d_{ij}^{\mu (n)}\right)}^{2}}$$ (31)

where *θ*^(*n*)^ is a smoothing parameter, which is initialized to 2 and halved if no improvement for the lower bound can be obtained in the consecutive 200 iterations. The denominator $\sum_{\forall i\in I}{\left(d_{i}^{\lambda (n)}\right)}^{2}+\sum_{\forall i\in I}\sum_{\forall j\in J}{\left(d_{ij}^{\mu (n)}\right)}^{2}$ denotes the norm of the move directions. Finally, we update the Lagrange multipliers $\lambda_{i}^{\left(n+1\right)}$ and $\mu_{ij}^{\left(n+1\right)}$ at the *n*th iteration by using the step size *t*^(*n*)^ and move directions $\left(d_{i}^{\lambda (n)},d_{ij}^{\mu (n)}\right)$ as follows:

$$\lambda_{i}^{\left(n+1\right)}=\max\left\{0,\lambda_{i}^{\left(n\right)}+t^{\left(n\right)}\cdot d_{i}^{\lambda (n)}\right\}$$ (32)

$$\mu_{ij}^{\left(n+1\right)}=\max\left\{0,\mu_{ij}^{\left(n\right)}+t^{\left(n\right)}\cdot d_{ij}^{\mu (n)}\right\}$$ (33)

Step 4: The algorithm stops if one of the following stop conditions is satisfied, and we hopefully obtain an optimal or nearly optimal solution. Otherwise, we increase *iterNum* by 1 and repeat step 2.

1. The gap between the upper bound and the lower bound is less than a predefined constant, i.e., $\frac{\left(UB-LB^{\left(n\right)}\right)}{UB}\leq \epsilon$. In this paper, we set *ϵ* = 0.0001.
2. *iterNum* reaches the maximal iteration number *N*. In this paper, we set *N* = 3000.
3. The stepsize *θ*^(*n*)^ is smaller than a predefined limit *θ*. In this paper, we set *θ* = 0.0001.

## Computational Experiments

We conducted a series of computational experiments to test the algorithm’s performance and gain some managerial insights from the model.

### Algorithm performance

To test the performance of the proposed Lagrangian relaxation algorithm and the effectiveness of the local search procedure, we compared the Lagrangian relaxation algorithm without local search (abbreviated as LR) and the Lagrangian relaxation algorithm with local search (abbreviated as LR+LS) with the commercial optimization solver CPLEX, version 12.6. All of the codes were written using C++, and CPLEX was invoked using Concert Technology. All experiments were run on the same SONY laptop with a Windows 7 64-bit operating system, an Intel Core i5 2.5 GHz CPU and 6.0 GB of physical RAM.

We tested the algorithms on four datasets that are commonly used in the reliable facility location literature: (1) a 49-node dataset that consists of 49 nodes representing the 48 state capitals of the continental United States plus Washington, D.C.; (2) an 88-node dataset that consists of the 49 cities in set (1) plus the 50 largest cities in the United States, minus duplicates; (3) a 150-node dataset that consists of the 150 largest cities in the United States; and (4) a 263-node dataset that consists of the 263 largest cities in the contiguous 48 states in the United States.

The fixed cost of the unreliable facility at site *j* is set as $f_{j}^{U}=500,000+1.7h_{i}$, which consists of a fixed cost and a variable cost proportional to the population at the node. The fixed cost of the reliable facility at site *j* is set as $f_{j}^{R}=f_{j}^{U}+5,000,000q_{j}$. For each node pair *i* and *j*, we compute their great circle distance *d*_*ij*_ according to their longitudes and latitudes and set $d_{ij}^{P}=0.02d_{ij}$. To reflect the fact that the emergency supply from a backup node is always more expensive than the regular supply, we set $d_{ij}^{B}=1.25d_{ij}^{P}$. To sufficiently test the performance of our approach for solving problems with site-specific probability, we randomly generate the failure probability *q*_*j*_ in interval [0, 0.05] using the uniform distribution.

Let *UB*(X) and *LB*(X) denote the minimal upper bound and maximal lower bound for algorithm X, respectively. Here, X refers to both LR and LR+LS. We use the performance gap and optimality gap to measure the performances of the algorithms, and they are defined as follows:

$$\text{Performance}\;\text{Gap}=\frac{UB(\text{X})-UB(\text{CPLEX})}{UB(\text{X})}*100$$ (34)

$$\text{Optimality}\;\text{Gap}=\frac{UB(\text{X})-LB(\text{X})}{UB(\text{X})}*100$$ (35)

For each data set, we randomly generate 20 instances with *q*_*j*_ ∈ *U*(0, 0.05). All of the data are available at S1 Dataset. We record the minimal, maximal, average value and standard deviation for the performance gap and optimality gap as well as the CPU time for each algorithm. The CPLEX is invoked with all of the default settings, and only the pure CPU time for the solver is recorded. The results are shown in Table 1.

**Table 1:** Algorithm performances for the four datasets.

Data set		Performance Gap (%)		Optimality Gap (%)		Computational time (seconds)		
		LR	LR+LS	LR	LR+LS	LR	LR+LS	CPLEX
49 nodes	Min.	0.00	0.00	0.01	0.01	<0.01	<0.01	37
	Max.	0.03	0.00	0.10	0.09	5	5	80
	Avg.	0.01	0.00	0.05	0.04	3.95	4.05	47.55
	Std.	0.01	0.00	0.03	0.02	1.70	1.61	9.73
88 nodes	Min.	0.00	0.00	0.07	0.09	32	32	340
	Max.	0.37	0.13	0.84	0.80	36	36	429
	Avg.	0.12	0.03	0.52	0.46	33.30	33.60	358.90
	Std.	0.12	0.04	0.26	0.21	1.08	1.05	23.17
150 nodes	Min.	0.00	0.00	0.15	0.36	271	273	1375
	Max.	0.58	0.24	1.15	1.05	292	301	4720
	Avg.	0.23	0.08	0.72	0.70	286.40	289.00	2974.10
	Std.	0.17	0.09	0.26	0.19	6.75	7.53	692.17
263 nodes	Min.	-	-	4.12	0.33	1896	1898	-
	Max.	-	-	14.24	1.13	1947	2045	-
	Avg.	-	-	9.08	0.78	1908.45	1941.75	-
	Std.	-	-	2.34	0.22	15.43	44.76	-

From Table 1, we can see that LR+LS performs very well for all four datasets and that it performs better than pure LR does with respect to both the performance gap and optimality gap. Additionally, both LR+LS and LR are far more efficient than CPLEX.

For the 49-node dataset, the LR+LS produces the same results as CPLEX, with a performance gap of 0. The LR also works well for this dataset with an average performance gap of 0.01%. Both algorithms have small optimality gaps, with average values of 0.05% and 0.04%, respectively. These values indicate that the both algorithms are very effective for small-sized problems.

For the 88-node dataset, LR+LS can still produce almost the same solutions as CPLEX; its worst performance gap is 0.13%, its average performance gap is only 0.03%, and the standard deviation is 0.04%. The pure LR obviously performs worse than LR+LS for this dataset, and its average performance gap is 0.12%.

For the 150-node dataset, the performance of pure LR deteriorates greatly, with the average performance gap increasing to 0.23%. LR+LS can still produce high-quality solutions, with an average performance gap of only 0.08% and a standard deviation of 0.09%, which illustrates that LR+LS works effectively for this dataset and obviously dominates pure LR. The optimality gaps of both algorithms increase, and LR+LS is slightly better than LR, with an average value of 0.7% compared to 0.72%.

CPLEX fails to solve the 263-node dataset due to insufficient memory; therefore, only the optimality gaps are recorded for the two algorithms for this dataset. The differences between the optimality gaps obtained for each of the algorithms are large for this dataset. More specifically, the average optimality gap of LR is 9.08%, whereas that of LR+LS is only 0.78%, which again illustrates the effectiveness of a local search for reducing the optimality gap. Because the performance gap seems to increase more slowly than the optimality gap, we can expect that LR+LS produces nearly optimal solutions for this large-sized dataset. To reduce the optimality gap, the Lagrangian relaxation algorithm can be embedded into a branch-and-bound framework, but it may require a longer computational time to produce an optimal or nearly optimal solution.

Considering the computational efficiency, it is easily seen that both LR and LR+LS are much more efficient than CPLEX. For all three of the datasets that can be solved using CPELX, the algorithms require less than 10% of the computational time required by CPLEX, illustrating that they are more than one order of magnitude faster than CPLEX. Moreover, the standard deviations of the computational time of the algorithms are much lower than those of CPLEX, especially for the 88- and 150-node datasets, which indicates that they are much more stable than CPLEX. We also observe from our experiments that the upper bound solution converges very quickly, taking far fewer than 3000 iterations to converge to the final solution, but the lower bound converges much more slowly; thus, the iteration limit is set to 3000 to increase the lower bound as far as possible. The computational time can be further reduced by setting the iteration limit to be a smaller number.

### Case study and managerial analysis

**Fig 2:**
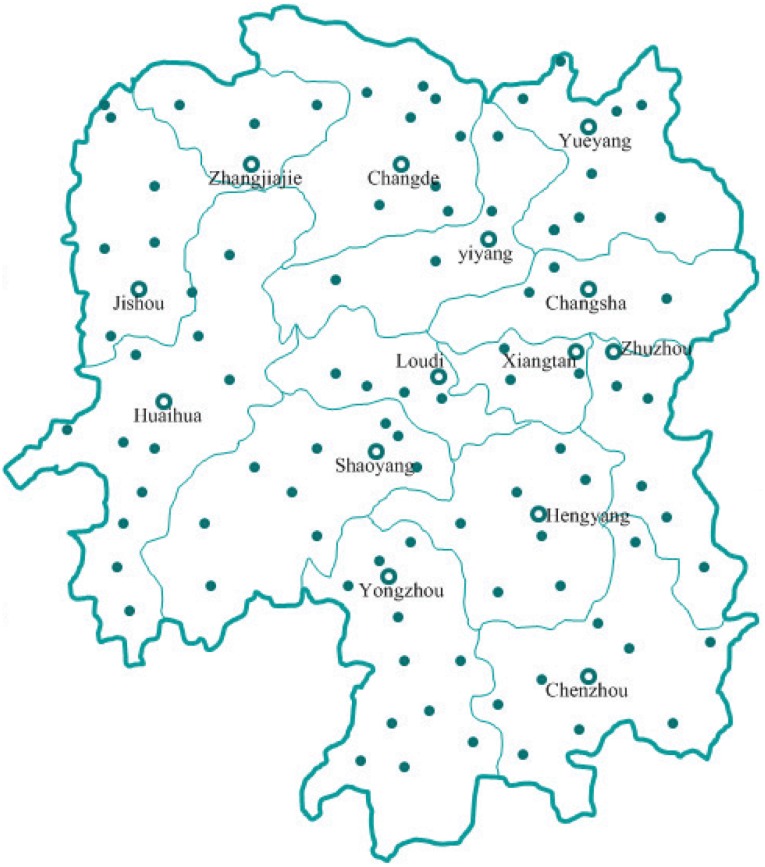
Demand points of the case example.

To analyze the model and obtain some managerial insights, we use the practical example of Hunan province to discuss the properties of the model. The case study contains the 100 main cities in Hunan province, as shown in Fig 2, which is located in the central south of China with an area of 211,800 *km*^2^ and a population of 71,193,400. Demand *h*_*i*_ is set as the population in the city according to the 2010 census data, divided by 100. The fixed cost of unreliable facilities is set as $f_{j}^{U}=500,000+2.5h_{i}$, and the fixed cost of reliable facilities is set as $f_{j}^{R}=f_{j}^{U}+5,000,000q_{j}$ to capture the fact that protecting a facility with a higher failure risk is more expensive. Again, for each node pair *i* and *j*, we compute their great circle distance *d*_*ij*_ according to their latitudes and longitudes, and we let $d_{ij}^{P}=d_{ij}$, $d_{ij}^{B}=1.25d_{ij}^{P}$. Data for this case example are available at S1 Dataset

The aim of these experiments is to analyze the impact of changes in different parameters on the optimal solution and different terms of cost. Three kinds of parameters are discussed in the following sections.

#### Impact of the facility failure probability

**Fig 3:**
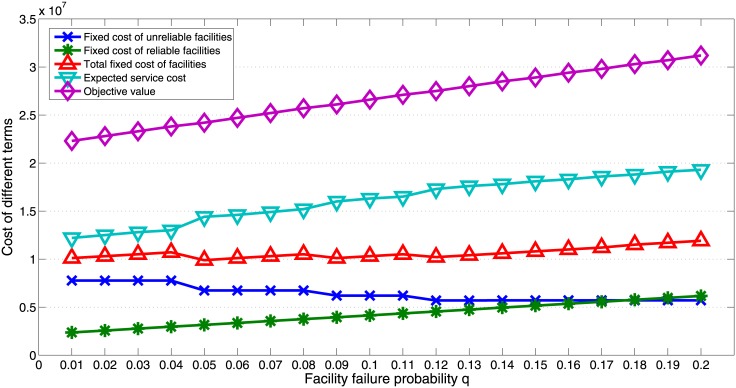
Cost corresponding to different facility failure probabilities q.

**Fig 4:**
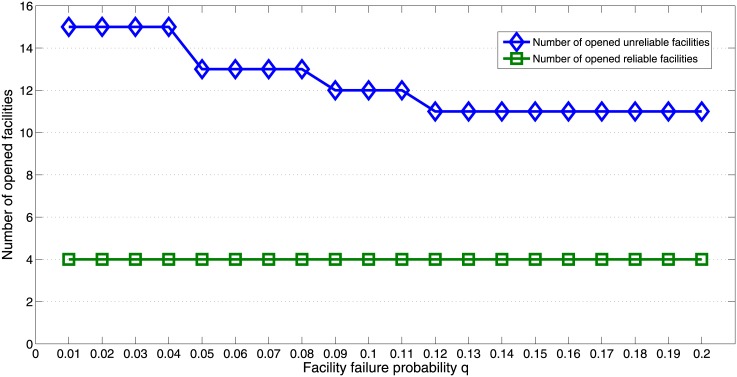
Number of opened unreliable and reliable facilities corresponding to different q.

Because the failure probability *q*_*j*_ is not easy to estimate precisely and is intrinsically uncertain, we attempt to understand how fluctuations in this parameter impact the optimal locations and different terms of cost. Here, we assume all *q*_*j*_ = *q* and let *q* fluctuate in the interval [0.01, 0.2]. We record different terms of cost and the number of opened facilities according to different *q*, which are presented in Figs 3 and 4.

As shown in Fig 3, the objective value seems to increase monotonously as *q* increases, and a similar trend can be observed from the curve of the expected service cost. This is not difficult to understand: as *q* increases, the unreliable facilities fail more frequently, and their customers must be rerouted to more expensive backup facilities more often, resulting in higher emergency service costs.

It is interesting to see that the fixed cost is insensitive to *q* because the number of opened unreliable facilities drops as *q* increases, while the number of reliable facilities remains steady. More specifically, as shown in Fig 4, when *q* ≤ 0.04, 15 unreliable facilities are opened, and when *q* ≥ 0.12, only 11 unreliable facilities are opened. The reason for this is obvious: though an unreliable facility is much cheaper than a reliable one, its failure may trigger additional emergency service costs due to customer rerouting. As *q* increases, the rerouting cost increases as well; thus, the number of unreliable facilities should be decreased.

**Fig 5:**
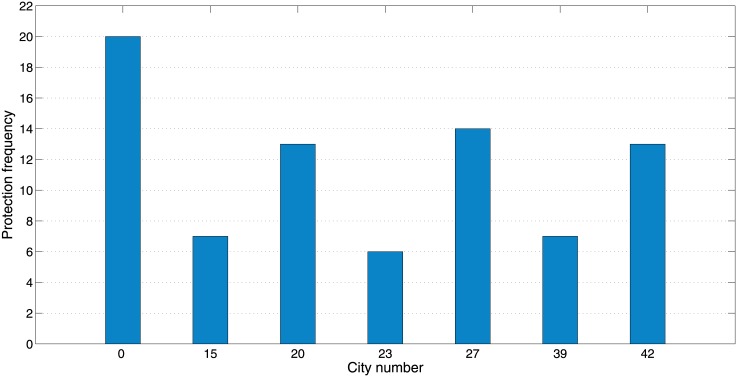
Frequency of opening a reliable facility for different sites.

At the same time, the number of opened reliable facilities remains fixed as *q* increases because the protection is expensive; thus, only a few of the most important cities can be protected. Specifically, when *q* changes from 0.01 to 0.2, only 7 of the 100 nodes appear to be among the cities that opened a reliable facility once, and their frequencies are shown in Fig 5. By carefully checking these nodes, we find that the optimal solution prefers to protect the largest cities or their satellite cities and transportation hubs. For example, node 0, which appears to be protected in all 20 cases, represents Changsha, the capital city of Hunan province, also the most populous city. Node 20 represents Changde, which is the largest city in western Hunan. Node 27 represents Xinshao, which is located approximately in the geographical center of Hunan and is an important transportation hub.

Table 2 shows the details of the opened facilities for different *q*, from which we can see that the optimal locations are similar or even the same as the others when *q* fluctuates slightly, which indicates that the optimal solution is robust to minor fluctuations in *q*. Therefore, although the facility failure probability is not easy to estimate precisely, the optimal solutions seem insensitive to minor fluctuations in the facility failure probability.

**Table 2:** The sites with facilities opened corresponding to different q.

q	sites with unreliable facilities opened	sites with reliable facilities opened
0.01-0.02	8,13,24,32,46,50,53,55,57,65,68,83,89,95,97	0,20,27,39
0.03-0.04	8,13,24,32,46,50,53,55,57,65,68,83,89,95,97	0,20,23,39
0.05-0.08	8,13,24,32,50,55,57,65,68,83,89,95,97	0,20,23,42
0.09-0.11	8,13,24,32,50,55,57,65,68,83,95,97	0,20,27,42
0.12-0.13	8,13,24,32,50,55,65,68,83,95,97	0,20,27,42
0.14-0.17	13,20,24,32,50,55,65,68,83,95,97	0,15,27,42
0.18-0.2	13,20,24,32,50,55,65,68,83,95,97	0,15,27,39,

In summary, when making location and protection decisions, the decision makers should account for the facility failure probability, demand and geographical position. Generally, when the failure probability is high, managers should open fewer unreliable facilities and open more reliable facilities to reduce the rerouting cost, and vice versa. At the same time, it seems better to centralize the protection of a few large cities or transportation hubs because these cities appear in the optimal solutions most frequently. Finally, the fixed cost seems insensitive to the facility failure probability; thus, even when the budget for fixed charges is limited, decision makers can still handle high disruption risks by adjusting the number of reliable and unreliable facilities.

#### Impact of the protection cost

Because $f_{j}^{R}=f_{j}^{U}+W\cdot q_{j}$, the protection cost is related not only to the failure probability but also to the protection cost coefficient *W*, which represents the cost for reducing the unit failure probability. The coefficient *W* is unrelated to the failure probability, but it is related to some other factors, such as the technical factors. Therefore, it may be relatively low under some circumstances but much higher under other circumstances. In this section, we discuss how the fluctuations of *W* affect the location and protection decisions.

**Fig 6:**
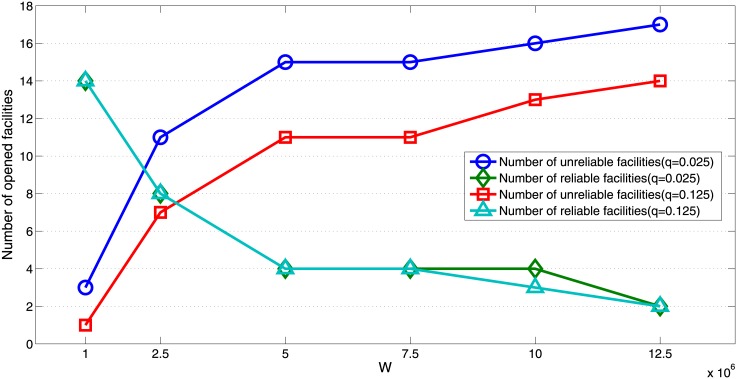
Number of sites with facilities opened corresponding to different W.

**Fig 7:**
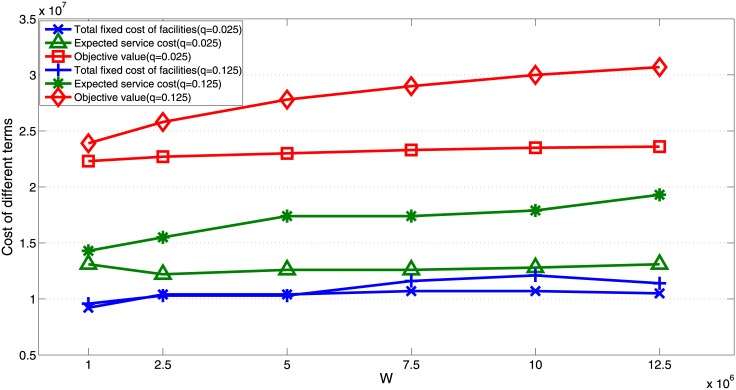
Costs corresponding to different W and two failure probabilities.

We let *W* vary from 1,000,000 to 12,500,000 and then observe its impact on the location decisions and the overall cost. At the same time, we set *q*_*j*_ = 0.025 and *q*_*j*_ = 0.125 to represent the cases when the failure probability is low and high, respectively, and we let $f_{j}^{R}=f_{j}^{U}+5,000,000q_{j}$. We record the number of opened facilities and different terms of cost corresponding to different *W* for both cases, which are depicted in Figs 6 and 7.

As can be seen from Fig 6, the numbers of both reliable and unreliable facilities are affected remarkably by *W*. As *W* increases, which means that protection becomes more expensive, fewer reliable facilities are opened; at the same time, more unreliable facilities are opened. This trend is especially obvious when *W* increases from 1,000,000 to 5,000,000. These observations are in accordance with our intuition: when the protection price is high, we should open fewer reliable facilities while opening more unreliable facilities, and vice versa.

Now, we observe the impact of *W* for different facility failure probabilities *q*. As can be seem from Fig 6, the numbers of reliable facilities when *q* = 0.025 and when *q* = 0.125 are very close in value. They are the same for the range of *W*, except when *W* = 10,000,000. In contrast, the number of unreliable facilities when *q* = 0.125 is much smaller than that when *q* = 0.025. This indicates that the number of reliable facilities is mainly affected by *W* but that the number of unreliable facilities is affected by both *W* and *q*.

As can be seen from Fig 7, both the overall cost and the service cost increase as *W* increases, and this trend is more obvious when the failure probability is high. More specifically, when *q* = 0.025, both the total cost and the service cost increase only slightly with *W*. For example, when *W* increases from 1,000,000 to 12,500,000, the total cost increases by 6.07% and the transportation cost increases by 0.23%. However, when *q* = 0.125, the objective value increases by 28.73% and the transportation cost increases by 35.17%. Therefore, if the failure probability is high, managers should try different ways of reducing the protection cost coefficient *W* because doing so can significantly impact both the service cost and the total cost; otherwise, they should not worry too much about a large *W*.

Compared to the service cost, the fixed cost seems much less sensitive to *W*, which holds true when *q* = 0.025 and *q* = 0.125. More specifically, as *W* increases from 1,000,000 to 12,500,000, i.e., it becomes 12.5 times larger, the fixed cost increases by only 14.32% when *q* = 0.025 and increases by only 19.13% when *q* = 0.125. The insensitivity of the fixed cost to *W* indicates that when the protection measures are expensive, decision makers can still obtain low-fixed-cost solutions, which can be achieved by reducing the number of reliable facilities opened and opening more unreliable facilities.

#### Impact of the emergency supply cost

The emergency supply cost coefficient $d_{ij}^{B}/d_{ij}^{P}$ is the ratio of the emergency supply cost to the regular cost for each unit demand, which reflects the system’s capability to cope with facility disruptions. A lower value denotes a better reactive emergency mechanism. The special case when $d_{ij}^{B}/d_{ij}^{P}=1$ represents the ideal situation, namely, the emergency cost is the same as the regular cost. However, this ideal situation may not be easy to ensure in reality, and we always have $d_{ij}^{B}/d_{ij}^{P}>1$. In this section, we discuss the impact of the emergency supply cost coefficient on the optimal solution and different terms of cost.

**Fig 8:**
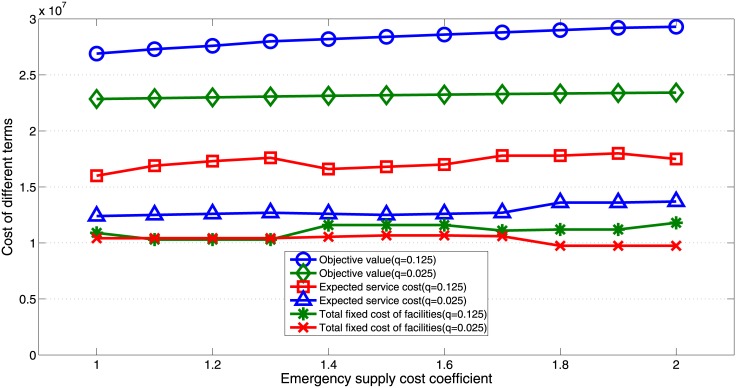
Costs of different terms corresponding to different $d_{ij}^{B}/d_{ij}^{P}$.

**Fig 9:**
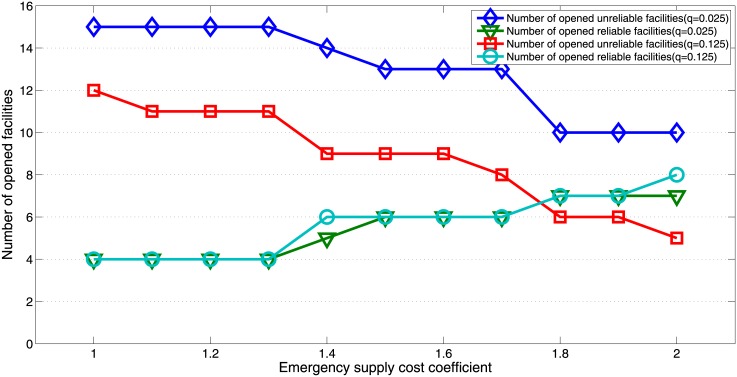
Numbers of opened facilities corresponding to different $d_{ij}^{B}/d_{ij}^{P}$.

We let $d_{ij}^{B}/d_{ij}^{P}$ increase from 1 to 2 to model the cases when the emergency supply becomes increasingly expensive, and we set *q* = 0.025 and *q* = 0.125 to represent the cases when the facility failure risk is low and high, respectively. The costs of different terms and the numbers of opened facilities according to different $d_{ij}^{B}/d_{ij}^{P}$ are presented in Figs 8 and 9.

Intuitively, one may think that the emergency supply cost coefficient affects the total cost significantly. Because the higher $d_{ij}^{B}/d_{ij}^{P}$ is, the more expensive it is to obtain an emergency supply, especially when the facility failure probability is high, the emergency supply will be triggered more frequently, which will result in higher emergency service costs. However, as shown in Fig 8, the objective value increases rather slowly as $d_{ij}^{B}/d_{ij}^{P}$ increases when both *q* = 0.025 and *q* = 0.125. More specifically, when $d_{ij}^{B}/d_{ij}^{P}$ becomes twice as large, the objective value increases by only 1.51% when *q* = 0.025 and by only 8.87% when *q* = 0.125.

Therefore, the overall cost is actually insensitive to $d_{ij}^{B}/d_{ij}^{P}$, at least when *q* ≤ 0.125. This seems counterintuitive at first glance, but it can be explained easily. When $d_{ij}^{B}/d_{ij}^{P}$ increases, the only increasing cost is the emergency service cost, which is both part of the total service cost and a small part of the total cost. Even when $d_{ij}^{B}/d_{ij}^{P}$ increases greatly, say to 2, and the facility failure probability is relatively high, say 0.125, assuming that the locations are the same, the increasing ratio of the emergency cost is still only 2 * 0.125 = 0.25, and the increasing ratio of the total cost is much smaller than 0.25 as the emergency cost is only part of it. This property is very useful because it reminds the decision makers that even when the emergency cost for each unit demand is high, they can still obtain cost-effective solutions by setting the number of both unreliable and reliable facilities wisely, as the objective value is actually insensitive to the emergency cost.

Fig 9 presents the number of opened facilities corresponding to different $d_{ij}^{B}/d_{ij}^{P}$. It can be easily seen that the number of unreliable facilities decreases and the number of reliable facilities increases as $d_{ij}^{B}/d_{ij}^{P}$ increases. This trend is more obvious when the facility failure probability is high. Therefore, when the emergency supply cost is high, the decision makers should open more reliable and fewer unreliable facilities to reduce the impact. By protecting more facilities, the overall cost will not be affected too greatly when the emergency service cost increases. However, the decision makers should also consider the protection cost and thus make good trade-offs.

## Conclusions

This paper proposes an integer programming model for the reliable facility location problem with facility protection, which allows for site-specific failure probabilities. The model uses both the proactive measure, i.e., protecting some of the facilities, and the reactive measure, i.e., obtaining an emergency supply from a backup facility when a customer’s primary facility fails. This facility protection and single-level backup mechanism can increase facility availability while reducing the operating complexities of facility networks.

An effective solution approach that combines Lagrangian relaxation and local search is developed to solve the model. Using numerical examples with different sizes, i.e., networks with 49, 88, 150 and 263 nodes, the performances of the proposed algorithms are compared with those of CPLEX, and the computational results show that our approach works well and consumes much less CPU time than CPLEX does for all of the examples.

Using a practical example from China, the influences of the facility disruption probabilities, protection cost and emergency service cost are analyzed, and we find that the overall cost increases as the facility failure probability and protection cost increase but is insensitive to the emergency service cost. Decision makers should wisely determine the number of both reliable and unreliable facilities to obtain a cost-effective solution.

In this work, all of the facilities are assumed to be uncapacitated, and a meaningful extension would be to consider the facility capacity constraints, which can make the problem reflect more practical situations. Additionally, as stated above, the facility failure probabilities are not easy to estimate precisely; thus, considering facility protection and backup assignment strategies in other modeling frameworks, such as robust optimization, may be a promising future direction. Finally, designing a facility network that is robust to both random disruptions and deliberate attacks is also worthy of further study.

## Supporting Information

S1 Dataset **The computational experiment dataset.** Includes the 49,88,150 and 263 nodes datasets, and each dataset contains 20 randomly generated instances. **The case example dataset.** Includes the information on the Hunan case example.
(ZIP)
