# Supplementary material for: Reliable Facility Location Problem with Facility Protection
Source: PLoS One. 2016 Sep 1;11(9):e0161532. doi: 10.1371/journal.pone.0161532 (PMC5008800; doi:10.1371/journal.pone.0161532)
Supplement: S1 Dataset — The computational experiment dataset. Includes the 49,88,150 and 263 nodes datasets, and each dataset contains 20 randomly generated instances. The case example dataset. Includes the information on the Hunan case example. (ZIP) [file pone.0161532.s001.zip › Supporting Information/QKCTVB5B_compare.pdf]

Reliable facility location problem with facility protection

Luohao Tang[1],Cheng Zhu[1,*], Zaili Lin[2], Jianmai Shi[1], Weiming Zhang[1]

1 Science and Technology on Information Systems Engineering Laboratory, National University of Defense Technology, Changsha, Hunan, CHINA
2 College of Mechanical and Power Engineering, Chongqing University of Science and Technology, Chongqing, CHINA

*** zhucheng@nudt.edu.cn**

Abstract

This paper studies a reliable facility location problem with facility protection ~~, which~~ that aims to hedge against random facility disruptions ~~through~~ by both strategically protecting some facilities and using backup facilities for the demands. An Integer Programming model is proposed for this problem~~is proposed~~, in which the failure probabilities of facilities are site-specific. A solution approach combining Lagrangian Relaxation and local search is proposed ~~, which is demonstrated~~ and is demonstrated to be both effective and efficient through computational experiments on random numerical examples with 49, 88, 150 and 263 nodes in the network~~respectively~~. A real case study for a 100-city network in Hunan province, China, is presented, based on which the properties of the model are discussed and some managerial insights are analyzed.

Author Summary

I am a PhD candidate ~~in~~ at the National University of Defense Technology, and my main research interests include developing mathematical models for analyzing ~~real life~~ real-life problems and designing efficient algorithms for hard combinatorial problems by using both mathematical ~~programming based~~ programming-based methods and heuristic methods. I was a visiting PhD student in the Department of Mechanical and Industrial Engineering of the University of Toronto during 2012.9-2014.9.

Introduction

Facilities are critical infrastructures of service networks and supply networks, and properly locating facilities is of great importance for providing products, information or services to ~~the~~ customers efficiently and sustainably. In real life, facilities may fail to work from time to time due to various disruptions, such as ~~earth quakes~~earthquakes, hurricanes, terrorist attacks, and equipment breakdowns. These disruptions substantially increase ~~the service cost as well as customer dissatisfactions, since customers should~~ both the service costs and customer dissatisfaction because customers may have to seek service elsewhere instead of their ~~preferable~~ preferred facilities or their demand ~~should~~ may be delayed or even ~~be~~ abandoned after a

disruption~~happened~~, which may lead to higher transportation costs, order delays, or a loss of market shares~~, and so on~~.

~~To make things worse~~Unfortunately, some disruptions may compromise the performance of the whole supply network and result in devastating consequences. For example, in 2001, an ~~eight minutes~~ eight-minute fire halted a ~~Philips's semiconductor factory~~ semiconductor factory belonging to Philips in New Mexico, USA, for 9 months, causing one of its customers, Ericsson, ~~lose~~ to lose USD 2.34 billion indirectly [?]. In 2011, a 8.9-magnitude earthquake and the ~~following~~ resulting tsunami struck Japan, which severely affected component plants of several industries~~,~~ ; production lines of many international companies were shut down for long ~~period~~ periods due to part ~~shortage~~ shortages from their Japanese suppliers [?, ?].

Facility location decisions are strategic~~,~~ ; once a facility network is constructed, it is costly and time consuming to reconfigure and rebuild. Additionally, recourses are always limited~~and restoration process~~ , and restoration processes can be very lengthy after a disputation~~happened. These all~~ . These factors highlight the need ~~to take~~ for taking facility disruptions into account and ~~design~~ designing a robust facility network ~~which owns some~~ that has the abilities to hedge against disruptions.

This paper studies a reliable facility location problem with facility protection, abbreviated as RFLPFP ~~hence, which~~ henceforth, that aims to increase the reliability of a facility network ~~through~~ by both protecting some facilities and assigning backup ~~facility~~ facilities for customers. More specifically, RFLPFP assumes that facilities may fail independently with site-specific failure probabilities ~~, but they~~ but can be protected to be reliable through extra investments. Different from the classical location models ~~which~~ that assign a customer to ~~exact~~ exactly one facility, in RFLPFP, if a customer is assigned to an unreliable facility, she should be assigned to a reliable facility ~~also~~as well, which works as her backup facility. Thus, the customer can ~~get emergency supply~~ obtain emergency supplies from her backup facility once her primary facility fails. This protection and backup mechanism ensures ~~all customers be served~~that all customers are served, even if some facilities fail~~down~~. The objective of RFLPFP is to determine the facility location and protection decisions as well as customer assignments to minimize the fixed charges and expected service cost.

This work contributes to the current literatures in terms of three main aspects. First, the facility location and protection problem is formulated ~~into~~ as an integer programming model where the site-specific failure probabilities are considered. Compared to ~~literatures~~ the literature on reliable facility location ~~problem~~ problems with facility protection, this new integer programming model more precisely captures the impact of different failure probabilities when facilities are located at different places. Second, a solution approach is developed ~~through~~ by combining Lagrangian Relaxation with some local search strategies. The performance of the approach is compared with the commercial optimization solver CPLEX ~~through~~ using random numerical examples with different sizes. Third, a practical case study is presented, based on which the impact of facility disruption probabilities, protection cost and emergency supply cost on the location decisions is analyzed.

The rest of this paper is organized as follows: in Section 2, some relevant ~~literatures~~ studies are reviewed. In Section 3, the RFLPFP problem is described, and a new linear integer programming model is proposed. In ~~section~~ Section 4, based on the formulation, a solution method combining Lagrangian Relaxation and local search is presented. In ~~section~~ Section 5, some computational experiments are conducted on some benchmark datasets, and comparisons of the developed algorithm and the CPLEX are ~~given. Also,~~ provided. Additionally, a sensitivity analysis is conducted on a case example, and some managerial insights are revealed. Finally, in Section 6, we ~~give~~ provide a conclusion and suggest several future research directions.

Literature review

~~Facility~~ The facility location problem is a classical optimization problem ~~and also~~ as well as a fundamental problem in designing supply or service ~~network~~networks, which has been extensively studied during the past decades. There ~~are abundant literatures~~ is an abundant body of literature on both deterministic and stochastic facility location problems [?, ?, ?, ?, ?]. However, a majority of the ~~literatures mainly deals with~~ literature mainly addresses demand and cost uncertainties [?], and relatively fewer studies consider the influence of facility disruptions. Most recently, both academics and practitioners ~~realize~~ have realized that facility disruptions may be triggered by various types of factors and may happen frequently, ~~there are more and more studies concerning~~ and there are an increasing number of studies on improving the reliability of facility networks through planning for facility disruptions [?, ?, ?].

~~Mainly, there are two~~ There are two main research streams on tackling ~~with~~ facility disruptions. The first one ~~tries~~ seeks to improve the availability of facilities ~~through~~ by increasing redundancy and utilizing backups. These models always explicitly consider the disruption probabilities when designing a facility network. Snyder et al. [?] study the reliable P-median problem and the reliable uncapacitated ~~fixed charge~~ fixed-charge location problem, which simultaneously optimize the operating cost under regular circumstances and the expected cost when disruption occurs. ~~Through analyzing the trade off curves of the both cost~~By analyzing the trade-off curves of both costs, they point out that substantial improvements of reliability can always be obtained ~~through only slight increase of~~ with only slight increases in the regular cost. Cui et al. [?] extend Snyder's work by relaxing the assumption of uniform disruption probability and allow site-specific disruption probabilities ~~,~~ and design a Lagrangian ~~relaxation based~~ relaxation-based algorithm as well as a continuous approximation algorithm to solve the problem. Berman et al. [?] study the reliable P-median problem on a network ~~,~~ and propose several exact and heuristic algorithms, which reveal that facilities become more centralized or even co-located as the failure probability increases. Shen et al. [?] propose a ~~scenario based~~ scenario-based stochastic program and a nonlinear integer program for the reliable facility location problem with ~~heterogonous~~ heterogeneous failure probabilities. They prove that the both models are equivalent and propose a constant-ratio approximation algorithm for the uniform case. Li et al. [?] consider the correlated effect of disruptions and propose a continuum approximation approach for the reliable facility location problem.

A common feature of the above papers is that they all utilize the ~~multiple level assignments~~ multiple-level assignment strategy to increase the reliability of a facility network~~. That~~ ; that is, each customer should be assigned to a group of facilities that are ordered by levels. Once a customer's n~~th level~~ th-level facility is disrupted, she will seek service from her $(n+1)$~~th level~~ th-level operational facility, and so on. Ultimately, a customer is served by an operational facility, or her demand is abandoned if all her assigned facilities fail~~, in the later case~~; in the latter case, a penalty is charged. ~~Even though~~ Though this multi-level backup mechanism can improve the availability of facilities, it also increases the operational and managerial complexities ~~since~~ because each customer has to ~~setup~~ set up connections with multiple facilities, and vice versa. A more complicated situation emerges when prior information on the operational states of facilities is unknown to a customer before reaching it, ~~so~~ and therefore, the customer may have to visit several disrupted facilities before getting an operational one [?, ?, ?].

The second stream of related ~~works~~ work focuses on improving facility availability through explicitly protecting or fortifying some of the most critical facilities. In reality, various protection measures are available, such as installation of structural reinforcements, adding built-in redundancies, improving monitoring and security

guarding, buying insurances or using outsourcing, and so on. Most of the papers considering facility protection assume a context of deliberate attacks, i.e., where an intelligent adversary intentionally tries to interdict the facility network to maximize the losses and where, in contrast, ~~by the contrary,~~ a defender protects some of the most critical components to mitigate the effect of the attacks.

Maria P. Scaparra et al. [?] study the r-interdiction median problem with fortification, which selects q facilities to protect among ~~existing~~ p existing facilities so that the impact of the most disruptive attack ~~to~~ on $r(q + r < p)$ unprotected facilities is minimized. Aksen et al. [?] study the protection ~~resource constrained~~ resource-constrained facility protection problems, in which the number of protected facilities is not predetermined but should be computed optimally while satisfying the protection resource constraints. Zhu et al. [?] further consider the probabilistic protection problem, which assumes that the probability of being interdicted for a facility decreases as more protection ~~resource is~~ resources are allocated to it. Liberatore et al. [?] consider the correlation between the facilities ~~,~~ and use a ~~two dimensional~~ two-dimensional correlation matrix to model the interdependence between facilities and present a location-attack-assignment ~~trilevel~~ tri-level model. A location-hardening problem with the objective of minimizing the maximum distance from a customer to its closest operational facility after facility disruptions is studied in [?]. Almost all of these protection models focus on the worst case, and no failure probability information about the facilities ~~are~~ is considered.

The objective of this paper is to combine both backup mechanism and facility protection to hedge against random facility disruptions. A few studies ~~we can find contributing on such kind~~ investigating such kinds of reliable facility location ~~problem are works presented by Lim 's work~~ problems are those by Lim et al. [?] and Li ~~'s work~~ et al. [?, ?]. Lim et al. [?] study a reliable facility location problem where facility fortification ~~option and single level~~ options and single-level backup strategies are adopted to improve the availability of the facilities. A linear integer programming model is presented to formulate the problem, and the efficiency of the model is illustrated through examples where all facilities have the same failure probability, but there is a lack of deep analysis for solving a problem with site-specific probabilities. Li et al. [?, ?] extend ~~Lim's work~~ the work of Lim et al. by further considering the protection budget constraint, and ~~make a~~ they make the strong assumption that a backup facility is always available even when it is not hardened. ~~Different form~~ Different from these works, this paper proposes a more general linear integer programming model ~~which~~ that can deal with ~~the~~ site-specific failure probabilities~~,~~; therefore, it is more suitable for the random disruptions triggered by natural or accidental events. Additionally, different ~~form~~ from Li's models, in our model, only the protected reliable facility can work as a backup facility, and a customer must be backed up to a protected facility if her primary facility is unreliable. This mechanism ensures ~~the customers always be~~ that customers are always served. Compared to the multi-level assignment ~~mechanism in existing literatures~~mechanisms in the existing literature, this paper adopts a ~~single level~~ single-level backup mechanism to hedge against facility disruptions, as ~~adopted~~ performed in [?, ?, ?], which makes the facility-customer relationship simpler and clearer ~~since~~ as each customer only needs to ~~setup~~ set up connections with at most two facilities, thus reducing the operational complexity of the system. Furthermore, the time latency for emergency ~~service~~ services can be reduced ~~since~~ because a backup facility is always available, which is critical, especially when the customers~~are time sensitive~~' needs are time-sensitive.

Mathematical formulation

RFLPFP is an extension of the classical facility location problem (FLP). It is assumed that facilities may fail independently with site-specific failure probabilities, and the probability information can be estimated ~~through analysis of~~ by analysis of the historical data. We also assume that facilities can be protected to be reliable through extra investments. Possible protection measures include built-in redundancies, structural reinforcements, preventive monitoring and safety guarding, ~~outsourcing, etc~~and outsourcing, among others. The protection resources are usually not sufficient to protect all facilities ~~into reliableones~~to become reliable. Therefore, there are two types of facilities in the system, the unreliable regular ones and the reliable protected ones. A customer should be assigned to a reliable facility directly if it is her nearest facility~~,~~ ; otherwise, she should be assigned to an unreliable facility as her primary facility and the nearest reliable facility as her backup facility. For the latter case, the customer can ~~get emergency service~~ obtain emergency services from her backup facility when her primary facility fails. This protection and backup mechanism ensures ~~each customer be served in probability~~ that each customer is served with a probability of 1. RFLPFP aims to determine the facility location and protection decisions as well as customer assignments to minimize the fixed charges and expected service cost.

To formulate the problem as an integer programming model, we use the following notations:

Notations:

I: set of customer points, indexed by i

J: set of potential facility sites, indexed by j

h_i: demand of customer i

q_j: failure probability of the unreliable facility opened at site j

f_j^U: fixed charge of opening an unreliable facility at site j

f_j^R: fixed charge of opening a reliable facility at site j, and $f_j^R > f_j^U$

d_{ij}^P: unit service cost when customer i is served by her primary facility opened at site j.

d_{ij}^B: unit service cost when customer i is served by her backup facility opened at site j. We assume $d_{ij}^B \geq d_{ij}^P$ to reflect that the emergency cost when customer i is served by her backup facility is ~~no~~ not lower than the regular cost when customer i is served by her primary facility.

Decision variables:

$X_j^R = 1$ ~~,~~ if a reliable facility is opened at site j; $X_j^R = 0$ ~~,~~ otherwise.

$X_j^U = 1$ ~~,~~ if an unreliable facility is opened at site j; $X_j^U = 1$ ~~,~~ otherwise.

$Y_{ikj} = 1$ ~~,~~ if customer i is assigned to an unreliable facility k as her primary facility and assigned to a reliable facility j as her backup facility; $Y_{ikj} = 0$ ~~,~~ otherwise.

$Z_{ij} = 1$ ~~,~~ if customer i is assigned to a reliable facility j as ~~its~~ her primary facility; $Z_{ij} = 0$ ~~,~~ otherwise.

The linear integer programming model for the RFLPFP is as follows.

$$(P1)\ \min \sum_{\forall j\in J} f_j^U X_j^U + \sum_{\forall j\in J} f_j^R X_j^R + \sum_{\forall i\in I}\sum_{\forall j\in J} h_i d_{ij}^P Z_{ij} + \sum_{\forall i\in I}\sum_{\forall k\in J, k\neq j}\sum_{\forall j\in J} (h_i d_{ik}^P (1-q_k) Y_{ikj} + h_i d_{ij}^B q_k Y_{ikj}) \tag{1}$$

$$s.t.$$

$$X_j^U + X_j^R \leq 1, \forall j \in J \tag{2}$$

$$\sum_{\forall k\in J, k\neq j}\sum_{\forall j\in J} Y_{ikj} + \sum_{\forall j\in J} Z_{ij} = 1, \forall i \in I \tag{3}$$

$$Z_{ij} \leq X_j^R, \forall i \in I, \forall j \in J \tag{4}$$

$$\sum_{\forall k\in J, k\neq j} Y_{ijk} \leq X_j^U, \forall i \in I, \forall j \in J \tag{5}$$

$$\sum_{\forall k\in J, k\neq j} Y_{ikj} \leq X_j^R, \forall i \in I, \forall j \in J \tag{6}$$

$$\sum_{\forall j\in J} X_j^R \geq 1 \tag{7}$$

$$X_j^R, X_j^U \in \{0,1\}, \forall j \in J \tag{8}$$

$$Z_{ij} \in \{0,1\}, \forall i \in I, \forall j \in J \tag{9}$$

$$Y_{ikj} \in \{0,1\}, \forall i \in I, \forall j, k \neq j \in J \tag{10}$$

The objective function ?? is to minimize the overall costs, including the fixed cost of opened unreliable and reliable facilities, the deterministic service cost for customers who are served by a reliable facility, and the expected service cost for customers who are served by a primary facility and a backup facility. Constraints ?? denote that either a reliable facility or an unreliable facility can be opened at a site, but not both. Constraints ?? state that a customer is either assigned to a reliable facility directly (if it is her nearest facility) or to an unreliable facility as her primary facility and a protected facility as her backup facility. Constraints ?? state that if a customer is assigned to only one layer facility, then the facility should be reliable. Constraints ?? and ?? represent that if a customer is assigned to two layer facilities, then the primary facility should be unreliable and the backup facility should be reliable, respectively. Constraint ?? states that there should be at least one reliable facility opened, which is a redundant constraint and can be derived through by combining constraints ??, ??, and ??, but we use it to tight tighten the bound when developing we develop the Lagrangian Relaxation algorithm. Constraints ??–?? are the integrality constraints.

Solution approach based on Lagrangian Relaxation

When all of the facilities have 0 failure probability a failure probability of 0, the RFLPFP is reduced to the classical uncapacitated fixed charged fixed-charge facility location problem, which is NP hard. Hence, RFLPFP is also NP hard. The model contains $2|J| + |I| \times |J| \times |J|$ binary variables and $|J| + |I| + 3|I| \times |J| + 1$ constraints. In this section, we present a Lagrangian Relaxation based Relaxation-based approach to solve the problem. The main ideas of the Lagrangian Relaxation approach are following: first, the "hard" constraints of the original problem are relaxed, which leads to a relaxation problem that can be solved relatively easily and help to obtain a low helps in obtaining a lower bound for the original problem. Then, based on the solution

to the relaxation problem, we can construct a feasible solution to the original problem, which provides an upper bound. Usually, some search algorithms can be used to improve the upper bound solution. ~~After that, the~~ The Lagrange multipliers are ~~adjusted towards~~ then adjusted for reducing the amount of ~~constraints~~ constraint violation, and ~~again, we~~ we again obtain a new relaxation problem corresponding to the updated Lagrange multipliers. This ~~procedure~~ procedure repeats until some stopping conditions are met. The details of the algorithm will be explained in the following subsections.

We relax constraints **??** with Lagrange multipliers λ and relax constraints **??** with Lagrange multipliers μ ~~,~~and obtain the corresponding Lagrangian relaxation problem.

$$Z_{LR}(\lambda,\mu\geq 0)=\min_{X^R,X^U,Z,Y}\sum_{\forall j\in J} f_j^U X_j^U+\sum_{\forall j\in J} f_j^R X_j^R+\sum_{\forall i\in I}\sum_{\forall j\in J} h_i d_{ij}^P Z_{ij}+$$
$$\sum_{\forall i\in I}\sum_{\forall k\in J,k\neq j}\sum_{\forall j\in J} h_i(d_{ik}^P(1-q_k)+d_{ij}^B q_k)Y_{ikj}+\sum_{\forall i\in I}\lambda_i(1-\sum_{\forall k\in J,k\neq j}\sum_{\forall j\in J} Y_{ikj}-\sum_{\forall j\in J} Z_{ij})$$
$$+\sum_{\forall i\in I}\sum_{\forall j\in J}\mu_{ij}(\sum_{\forall k\in J,k\neq j} Y_{ijk}-X_j^U)$$
$$s.t. ??, ??, ??, ??, ??, ??, ?? \quad (11)$$

~~Since~~ Because $\sum_{\forall i\in I}\sum_{\forall j\in J}\sum_{\forall k\in J,k\neq j}\mu_{ij}Y_{ijk}=\sum_{\forall i\in I}\sum_{\forall k\in J}\sum_{\forall j\in J,j\neq k}\mu_{ik}Y_{ikj}=\sum_{\forall i\in I}\sum_{\forall k\in J,k\neq j}\sum_{\forall j\in J}\mu_{ik}Y_{ikj}$, the above formulation can be rewritten as:

$$Z_{LR}(\lambda,\mu\geq 0)=\min_{X^R,X^U,Z,Y}\sum_{\forall j\in J} f_j^R X_j^R+\sum_{\forall j\in J}(f_j^U-\sum_{\forall i\in I}\mu_{ij})X_j^U+\sum_{\forall i\in I}\sum_{\forall j\in J}(h_i d_{ij}^P-\lambda_i)Z_{ij}$$
$$+\sum_{\forall i\in I}\sum_{\forall k\in J,k\neq j}\sum_{\forall j\in J}(h_i d_{ik}^P(1-q_k)+h_i d_{ij}^B q_k+\mu_{ik}-\lambda_i)Y_{ikj}+\sum_{\forall i\in I}\lambda_i$$
$$s.t. ??, ??, ??, ??, ??, ??, ?? \quad (12)$$

For the sake of brevity, we rewrite the above formulation **??** as follows:

$$Z_{LR}(\lambda,\mu\geq 0)=\min_{X^R,X^U,Z,Y}\sum_{\forall j\in J} f_j^R X_j^R+\sum_{\forall j\in J}\alpha_j X_j^U+\sum_{\forall i\in I}\sum_{\forall j\in J}\beta_{ij}Z_{ij}+$$
$$\sum_{\forall i\in I}\sum_{\forall k\in J,k\neq j}\sum_{\forall j\in J}\gamma_{ikj}Y_{ikj}+\sum_{\forall i\in I}\lambda_i$$
$$s.t. ??, ??, ??, ??, ??, ??, ?? \quad (13)$$

where $\alpha_j=f_j^U-\sum_{\forall i\in I}\mu_{ij}$, $\beta_{ij}=h_i d_{ij}^P-\lambda_i$, and $\gamma_{ikj}=h_i d_{ik}^P(1-q_k)+h_i d_{ij}^B q_k+\mu_{ik}-\lambda_i$.

For given Lagrange multipliers λ and μ, **??** provides a lower bound for the original problem **??**. ~~And the~~ The Lagrangian dual problem ~~is to find~~ involves finding the maximal lower bound of the original problem **??** ~~through~~ by calculating the optimal Lagrange multipliers λ and μ, which can be expressed as follows:

$$LD(\lambda,\mu\geq 0)=\max_{\lambda,\mu\geq 0}\min_{X^R,X^U,Z,Y}\sum_{\forall j\in J} f_j^R X_j^R+\sum_{\forall j\in J}\alpha_j X_j^U+\sum_{\forall i\in I}\sum_{\forall j\in J}\beta_{ij}Z_{ij}+$$
$$\sum_{\forall i\in I}\sum_{\forall k\in J,k\neq j}\sum_{\forall j\in J}\gamma_{ikj}Y_{ikj}+\sum_{\forall i\in I}\lambda_i$$
$$s.t. ??, ??, ??, ??, ??, ??, ?? \quad (14)$$

The dual problem **??** can be solved by using the sub-gradient algorithm [?, ?].

Solving the Lagrangian relaxation problem

A very crucial step is to solve the relaxation problem, that is, to solve the minimization problem **??** for the given Lagrange multipliers λ and μ.

~~Notice that if we ignore the~~ If we ignore constraint **??**, which ~~restricts~~ stipulates the restriction that at least one reliable facility ~~be~~ is opened, the Lagrangian relaxation problem $Z_{LR}(\lambda, \mu \geq 0)$ is separable on j, and it can be decomposed into $|J|$ independent ~~sub problems~~ subproblems, which can be solved relatively easily. Specifically, the ~~sub problem~~ subproblem corresponding to site j while dropping **??** can be expressed as shown below:

$$(P_j) \quad \min f_j^R X_j^R + \alpha_j X_j^U + \sum_{\forall i \in I} \beta_{ij} Z_{ij} + \sum_{\forall i \in I} \sum_{\forall k \in J, k \neq j} \gamma_{ikj} Y_{ikj} \tag{15}$$

$$s.t.$$

$$X_j^U + X_j^R \leq 1 \tag{16}$$

$$Z_{ij} \leq X_j^R, \forall i \in I \tag{17}$$

$$\sum_{\forall k \in J, k \neq j} Y_{ikj} \leq X_j^R, \forall i \in I \tag{18}$$

$$X_j^R, X_j^U \in \{0, 1\} \tag{19}$$

$$Z_{ij} \in \{0, 1\}, \forall i \in I \tag{20}$$

$$Y_{ikj} \in \{0, 1\}, \forall i \in I, \forall k \neq j \in J \tag{21}$$

For each site j, there are three possible states, i.e., an unreliable facility is opened, a reliable facility is opened, and no facility is opened~~, respectively~~. We first compute the objective value **??** corresponding to each state~~, and~~ ; then, the state with the ~~objective value minimized~~ minimum objective value will be selected, and values for the decision variables $(X_j^U, X_j^R, Z_{ij}, Y_{ikj})$ will be set according to the selected state. Details for these three cases are ~~as~~ presented below.

Case 1: assuming that $X_j^U = 1$, which means that one unreliable facility is opened at j. ~~Denoting~~ We denote the objective value for this case as V_j^U . ~~Since~~ Because when $X_j^U = 1$, we have $X_j^R = 0, Z_{ij} = 0, \forall i \in I, Y_{ikj} = 0, \forall i \in I, \forall k \neq j \in J$, it can be easily seen from expression **??** that $V_j^U = \alpha_j$.

Case 2: assuming that $X_j^R = 1$, which means that one reliable facility is built at j. This case is more complicated in comparison to case 1. ~~Denoting~~ We denote the objective value for this case as V_j^R. When $X_j^R = 1$, according to constraints **??-??**, we can derive that $X_j^U = 0, Z_{ij} \leq 1, \forall i \in I$, and $\sum_{\forall k \in J, k \neq j} Y_{ikj} \leq 1, \forall i \in I$. ~~Based on these, we~~ We then obtain $V_j^R = f_j^R + \sum_{\forall i \in I} \min(\beta_{ij}, 0) + \sum_{\forall i \in I} \min(\min_{\forall k \in J, k \neq j} \gamma_{ikj}, 0)$, and the ~~value~~ values for Z_{ij} and Y_{ikj} are set as follows:

$$Z_{ij} = \begin{cases} 1, & \text{if } \beta_{ij} < 0; \\ 0, & \text{otherwise.} \end{cases}, \forall i \in I. \tag{22}$$

$$Y_{ikj} = \begin{cases} 1, & \text{if } \gamma_{ikj} < 0 \text{ and } k = \text{argmin}_{\forall k \in J, k \neq j} \gamma_{ikj}; \\ 0, & \text{otherwise.} \end{cases}, \forall i \in I, \forall k \in J, k \neq j. \tag{23}$$

Case 3: assuming that $X_j^U = X_j^R = 0$, which means that no facility is built on site j. In this case, we can easily derive that $Z_{ij} = 0, \forall i \in I, Y_{ikj} = 0, \forall i \in I, \forall k \neq j \in J$. ~~So~~Therefore, its contribution to the objective function is 0.

Next, still ignoring constraint **??**, we determine the state for every site j ~~, we determine its state~~ as follows: If $V_j^U = \min(V_j^U, V_j^R, 0) < 0$, we set $X_j^U = 1$, and the

values for variables (X_j^R, Z_{ij}, Y_{ikj}) associated with j are set as stated for case 1. If $V_j^R = \min(V_j^U, V_j^R, 0) < 0$, we set $X_j^R = 1$, and the values for (X_j^U, Z_{ij}, Y_{ikj}) are set as stated for case 2. If $\min(V_j^U, V_j^R, 0) = 0$, we set all of the variables $(X_j^R, X_j^U, Z_{ij}, Y_{ikj})$ as 0.

~~Now, we take constraint ??in to account~~We now consider constraint **??**. Let $J^R = \{j | X_j^R = 1, j \in J\}$ denote the set of sites at which a reliable facility is opened, let $J^U = \{j | X_j^U = 1, j \in J\}$ denote the set of sites at which an unreliable facility is opened, and let $J^C = J \backslash \{J^U, J^R\}$ denote the rest of the sites at which no facility has been opened yet. If we have at least one reliable facility ~~built~~opened, i.e., $|J^R| \geq 1$, then the ~~computation of the~~ lower bound has been ~~done, since~~ achieved because the constraint **??** has been satisfied already. Otherwise, we need to open a reliable facility at a site in either J^U or J^C. We determine the best location for opening a reliable facility as follows:

Let $VJ_j^U = V_j^R - V_j^U, j \in J^U$ and $VJ_j^C = V_j^R, j \in J^C$. If $\min_{j \in J^U} VJ_j^U < \min_{j \in J^C} VJ_j^C$, we set $X_{j^*}^U = 1$, where $j^* = argmin_{j \in J^U} VJ_j^U$~~,~~; otherwise, we set $X_{j^*}^R = 1$, where $j^* = argmin_{j \in J^C} VJ_j^C$.

Once the location j^* for opening the reliable facility is determined, we set the values for the other decision variables $(X_{j^*}^U, Z_{ij^*}, Y_{ikj^*})$ associated with j according to case 2 as specified before.

~~Through~~ By solving the Lagrangian relaxation problem, we ~~get~~ obtain the lower bound:

$$Z_{LR}(\lambda, \mu \geq 0) = \sum_{\forall j \in J} (V_j^U X_j^U + V_j^R X_j^R) + \sum_{\forall i \in I} \lambda_i. \tag{24}$$

Computation of the upper bound

After solving the Lagrangian relaxation problem, we obtain a feasible location configuration (X_j^U, X_j^R), which may not be the optimal location configuration for the original problem **??** ~~, but it~~ but can be used to construct a feasible solution ~~which~~ that provides an upper bound of **??**. We let the location decisions of the feasible solution be the same as that of the relaxation problem, and the customer assignment of the feasible solution is the optimal assignment corresponding to the location configuration, which is computed as described below.

~~Still, let~~ Let J^R be the set of opened reliable facilities, and let J^U be the set of opened unreliable facilities. First, we ~~give~~ provide some properties:

Property 1: If customer i has a reliable facility j^* as her backup facility, then ~~,~~ $j^* = argmin_{j \in J^R} d_{ij}^B$.

Proof: Assume that i has an unreliable facility $k \in J^U$ as her primary facility and another reliable facility $j^\circ \in J^R, j^\circ \neq j^*$ as her backup facility~~, then~~; then, the expected service cost for i is $h_i d_{ik}^P (1 - q_k) + h_i d_{ij^\circ}^B q_k$. As $j^* = argmin_{j \in J^R} d_{ij}^B$, we have $h_i d_{ik}^P (1 - q_k) + h_i d_{ij^\circ}^B q_k \geq h_i d_{ik}^P (1 - q_k) + h_i d_{ij^*}^B q_k$~~,~~; hence, it is better to have j^* as her backup facility.

Property 2: Let $j^* = argmin_{j \in J^R} d_{ij}^B$~~,~~; if customer i has an unreliable facility k^* as her primary facility, then ~~,~~ $k^* = argmin_{k \in J^U} (d_{ik}^P (1 - q_k) + d_{ij^*}^B q_k)$.

Proof: Assume i has another unreliable facility $l \in J^U, l \neq k$ as her primary facility ~~,~~ and that the expected service cost for i is $h_i d_{il}^P (1 - q_l) + h_i d_{ij^*}^B q_l$. As $k^* = argmin_{k \in J^U} (d_{ik}^P (1 - q_k) + d_{ij^*}^B q_k)$, we have $h_i d_{il}^P (1 - q_l) + h_i d_{ij^*}^B q_l \geq h_i d_{ik^*}^P (1 - q_{k^*}) + h_i d_{ij^*}^B q_{k^*}$~~,~~; hence, it is better to have k^* as her primary facility.

Property 3: Let $j^* = argmin_{j \in J^R} d_{ij}^B$, $k^* = argmin_{k \in J^U} (d_{ik}^P (1 - q_k) + d_{ij^*}^B q_k)$, and $j^\circ = argmin_{j \in J^R} d_{ij}^P$~~, then,~~. Then, if $d_{ij^\circ}^P \leq (d_{ik^*}^P (1 - q_{k^*}) + d_{ij^*}^B q_{k^*})$, customer i

should be assigned to j° directly~~,~~ ; otherwise, she should be assigned to k^* as her primary facility and j^* as her backup facility.

Proof: Customer i is either assigned to a reliable facility directly ~~,~~ or assigned to an unreliable facility as her primary facility and a reliable facility as her backup facility. For the former case, the service cost is $h_i d_{ij^\circ}$~~,~~ ; for the latter case, the service cost is $h_i(d^P_{ik^*}(1-q_{k^*}) + d^B_{ij^*}q_{k^*})$. Obviously, the assignment with smaller service cost will be selected.

Based on the above properties, the procedure ~~to determine~~ for determining the optimal assignment for customer i is straightforward ~~, which can be done~~ and can be performed in $O(|J|)$ as follows:

First, we compute $j^\circ = argmin_{j\in J^R} d^P_{ij}$, $j^* = argmin_{j\in J^R} d^B_{ij}$, and $k^* = argmin_{k\in J^U}(d^P_{ik}(1-q_k) + d^B_{ij^*}q_k)$.

Then, if $d^P_{ij^\circ} \le d^P_{ik^*}(1-q_{k^*}) + d^B_{ij^*}q_{k^*}$, we set

$$Z_{ij} = \begin{cases} 1, & \text{if } j = j^\circ; \\ 0, & \text{otherwise.} \end{cases}, \text{and } Y_{ikj} = 0, \forall k, \forall j \in J. \tag{25}$$

~~otherwise~~Otherwise, we set

$$Y_{ikj} = \begin{cases} 1, & \text{if } k = k^* \text{ and } j = j^*; \\ 0, & \text{otherwise.} \end{cases}, \text{and } Z_{ij} = 0, \forall j \in J. \tag{26}$$

The objective value ~~corresponded~~ corresponding to this feasible solution is

$$\sum_{\forall j\in J}(f^U_j X^U_j + f^R_j X^R_j) + \sum_{\forall i\in I}\min(h_i d^P_{ij^\circ}, h_i d^P_{ik^*}(1-q_{k^*}) + h_i d^B_{ij^*}q_{k^*}), whichisanupperboundfortheo$$

Local search to improve the upper bound solution

As commonly used in Lagrangian ~~Relaxation based~~ Relaxation-based algorithms, a local search procedure is designed to improve the upper bound solution, which is demonstrated to be both effective and efficient ~~in later~~ based on the computational experiments.

The local search is based on switching states of the sites. Specifically, each site has ~~and only has~~ only three possible states as stated before~~, no facility is openedthere~~: no facility opened, denoted as 0; one unreliable facility ~~is openedthere~~opened, denoted as 1; and one reliable facility ~~is openedthere~~opened, denoted as 2. Hence, ~~to~~ for each site, there are two possible moves, i.e.~~switching from her~~ , switching from the current state to the other two states. It should be noticed that when a site ~~with~~ that has a reliable facility opened switches its state, i.e., 2→0 or 2→1, it should be guaranteed that at least one reliable facility remains opened in the system. For each move, the customer assignment and objective value corresponding to the new location configuration can be computed by using the method stated before. An illustrative example for the local moves is depicted in Fig. ??, where the left graph shows the initial location and assignment configurations, the middle one shows the configurations when site 2 moves from state 1 to state 2, and the right graph shows the configurations when site 2 moves from state 1 to state 0.

Figure 1. Illustrative example for the local moves.

We use a steepest descent strategy to choose the moves. For each inner loop, the move with the ~~most cost saving~~ highest cost savings will be conducted, and this

$$\text{Optimality Gap} = \frac{UB(\text{X}) - LB(\text{X})}{UB(\text{X})} * 100 \quad (35)$$

For each data set, we randomly generate 20 instances with $q_j \in U(0, 0.05)$~~, all the~~. All data are available at ??. We record the minimal, maximal, average value and standard deviation for the performance gap ~~,~~ and optimality gap as well as the CPU time for each algorithm. The CPLEX is invoked with all ~~the settings default~~ of the default settings, and only the pure CPU time for the solver is recorded. The results are shown in Table ??.

Table 1. Algorithm performances for the four datasets.

Data set		Performance Gap (%)		Optimality Gap (%)		Computational time (seconds)		
		LR	LR+LS	LR	LR+LS	LR	LR+LS	CPLEX
49 nodes	Min.	0.00	0.00	0.01	0.01	<0.01	<0.01	37
	Max.	0.03	0.00	0.10	0.09	5	5	80
	Avg.	0.01	0.00	0.05	0.04	3.95	4.05	47.55
	Std.	0.01	0.00	0.03	0.02	1.70	1.61	9.73
88 nodes	Min.	0.00	0.00	0.07	0.09	32	32	340
	Max.	0.37	0.13	0.84	0.80	36	36	429
	Avg.	0.12	0.03	0.52	0.46	33.30	33.60	358.90
	Std.	0.12	0.04	0.26	0.21	1.08	1.05	23.17
150 nodes	Min.	0.00	0.00	0.15	0.36	271	273	1375
	Max.	0.58	0.24	1.15	1.05	292	301	4720
	Avg.	0.23	0.08	0.72	0.70	286.40	289.00	2974.10
	Std.	0.17	0.09	0.26	0.19	6.75	7.53	692.17
263 nodes	Min.	-	-	4.12	0.33	1896	1898	-
	Max.	-	-	14.24	1.13	1947	2045	-
	Avg.	-	-	9.08	0.78	1908.45	1941.75	-
	Std.	-	-	2.34	0.22	15.43	44.76	-

From Table ??, we can see that ~~the~~ LR+LS performs very well for all ~~the~~ four datasets and that it performs better than ~~the~~ pure LR with ~~regard~~ respect to both the performance gap and optimality gap. Additionally, both LR+LS and LR are far more efficient than ~~the~~ CPLEX.

For the 49 nodes dataset, the LR+LS produces the same results as ~~the~~ CPLEX, with ~~performance gap equaling~~ the performance gap being 0. The LR also works well for this dataset with an average performance gap of 0.01%. Both algorithms have small optimality gaps, with average ~~value~~ values of 0.05% and 0.04%, respectively. These indicate the both ~~developed~~ algorithms are very effective for ~~small sized problem~~small-sized problems.

For the 88 nodes dataset, ~~the~~ LR+LS can still produce almost the same solutions as CPLEX~~, even~~ ; its worst performance gap ~~reaches~~ is 0.13%, its average performance gap is only 0.03%~~and the~~ , and the standard deviation is 0.04%. The pure LR obviously performs worse than LR+LS for this dataset, and its average performance gap ~~reaches~~ is 0.12%.

For the 150 nodes dataset, the performance of ~~the~~ pure LR deteriorates a lot, with the average performance gap increasing to 0.23%~~, but the~~ . LR+LS can still produce ~~high quality solutionswith~~ high-quality solutions, with an average performance gap of only 0.08% and ~~deviation~~ standard deviation of 0.09%, which illustrates that ~~the~~ LR+LS works effectively for this dataset and ~~it obviously dominates the~~ obviously dominates pure LR. ~~Both algorithms' optimality gaps increase~~ The optimality gaps of both algorithms increase, and LR+LS is slightly better than ~~the~~ LR, with an average value of 0.7% ~~comparing~~ compared to 0.72%.

CPLEX fails to solve the ~~instances of~~ 263 nodes dataset due to insufficient memory~~,~~ ; therefore, only the optimality gaps are recorded for the two algorithms for this dataset. The differences between the optimality gaps of the two algorithms are ~~big~~ large for this dataset. More specifically, the average optimality gap of ~~the LR reaches~~ LR is 9.08%, while that of the LR+LS is only 0.78% , which again illustrates the effectiveness of ~~the local search on~~ a local search for reducing the optimality gap. ~~Since~~ Because the performance gap seems to increase ~~much~~ more slowly than the optimality gap, we can expect ~~the~~ that LR+LS ~~produce nearly optimal solution~~ produces nearly optimal solutions for this large-sized dataset. To reduce the optimality gap, the ~~developed Lagraigian~~ Lagrangian Relaxation algorithm can be embedded into a ~~branch and bound~~ branch-and-bound framework, but it may ~~take much longer computation time to get~~ require a longer computational time to produce an optimal or nearly optimal solution.

Considering the computational efficiency, it is easily seen that both LR and LR+LS are much more efficient than ~~the~~ CPLEX. For all ~~the three datasets solvable for CPELX, the~~ three datasets that can be solved using CPELX, both algorithms require less than 10% ~~computation time of the~~ of the computational time required by CPLEX, illustrating that they are more than one order of magnitude faster than ~~the~~ CPLEX. Moreover, the standard deviations of ~~computation~~ the computational time of the ~~developed~~ algorithms are much ~~less than that~~ lower than those of CPLEX, especially for the 88 and 150 nodes ~~dataset, indicating~~ datasets, indicating that they are much more stable than CPLEX. ~~Actually, we also observe in~~ We also observe from our experiments that the upper bound solution converges very quickly, ~~and it takes~~ taking much less than 3000 iterations to converge to the final solution, but the lower bound converges much ~~slowly, thus~~more slowly; thus, the iteration limit is set to 3000 to increase the lower bound as far as possible. The ~~computing~~ computational time can be further reduced by setting the iteration limit to be a smaller number.

Case study and managerial analysis

To analyze the model and gain some managerial insights, we use ~~a~~ the practical example of the Hunan province to discuss the properties of the model. The case study contains the 100 main cities in Hunan province, as shown in Fig. ??, which ~~locates~~ is located in the central south ~~China with~~ of China with an area of 211,800 ~~kilometers areas and~~ km and a population of 71,193,~~400 populations.~~ 400. Demand h_i is set as the population in the city according to the 2010 census data, divided by 100. ~~Fixed~~ The fixed cost of unreliable facilities is set as $f_j^U = 500,000 + 2.5h_i$ ~~, and~~ and the fixed cost of reliable facilities is set as $f_j^R = f_j^U + 5,000,000q_j$ ~~,~~ to capture the fact that protecting a facility with a higher failure risk is more expensive. Again, for each node pair i and j, we compute their great circle distance d_{ij} according to their latitudes and longitudes, and we let $d_{ij}^P = d_{ij}$, $d_{ij}^B = 1.25d_{ij}^P$. Data ~~about~~ on this case example are available at **??**

Figure 2. Demand points of the case example.

The aim of these experiments is to analyze the impact of changes ~~of~~ in different parameters on the optimal solution and different terms of cost. Three kinds of parameters are discussed in the following sections.

Impact of the facility failure probability. ~~Since~~ Because the failure probability q_j is not easy to estimate precisely ~~, and it~~ and is intrinsically uncertain, we ~~try~~ attempt to understand how ~~the fluctuations of~~ fluctuations in this parameter impact

the optimal locations and different terms of cost. Here, we assume all $q_j = q$ and let ~~the~~ q ~~fluctuates in~~ fluctuate in the interval $[0.01, 0.2]$. We record different terms of cost and the number of opened facilities according to different q, which are ~~depicted~~ presented in Fig. ?? and Fig. ??.

Figure 3. Cost corresponding to different facility failure ~~probability~~ probabilities q.

Figure 4. Number of opened unreliable and reliable facilities corresponding to different q.

As can be seen from Fig. ??, the objective value seems to increase monotonously as q increases, and a similar trend can also be observed ~~on~~ from the curve of the expected service cost. This is not hard to understand~~,~~: as q increases, the unreliable facilities fail more ~~frequent,~~ frequently, and their customers have to ~~reroute~~ be rerouted to more expensive backup facilities more often, resulting in higher emergency service ~~cost~~costs.

It is interesting to see that the fixed cost is insensitive to q ~~, this is~~ because the number of opened unreliable facilities drops as q increases while the number of reliable facilities ~~keeps~~ remains steady. More specifically, as can be seen from Fig. ??, when $q \leq 0.04$, 15 unreliable facilities are opened, and when $q \geq 0.12$, only 11 unreliable facilities are opened. The reason for this is obvious~~, even~~ : though an unreliable facility is much cheaper than a reliable one, its failure may trigger additional emergency service ~~cost~~ costs due to customer rerouting. As q ~~becomes larger~~increases, the rerouting cost ~~becomes higher too, thus~~increases as well; thus, the number of unreliable facilities should be ~~reduced~~decreased.

At the same time, the number of opened reliable facilities ~~keep~~ remains fixed as q increases ~~, this is~~ because the protection is expensive~~and thus~~; thus, only a few of the most important cities can be protected. Specifically, when q changes from 0.01 to 0.2, only 7 of the 100 nodes appear to be among the cities ~~once opening~~ that opened a reliable facility ~~,~~ once, and their frequencies are shown in Fig. ??. ~~Through~~ By carefully checking these nodes, we find that the optimal solution prefers to protect the largest cities or their satellite cities ~~, and the~~ and transportation hubs. For example, node 0, which appears to be protected in all ~~the~~ 20 cases, represents Changsha, the capital city of Hunan province~~and~~ , also the most populous city. Node 20 represents Changde, which is the largest city in western Hunan. Node 27 represents Xinshao, which is located approximately in the geographical center of Hunan ~~,~~ and is an important transportation hub.

Figure 5. Frequency of opening a reliable facility for different sites.

Table ?? shows the details of the opened facilities for different q, from which we can see that the optimal locations are similar or even the same ~~with each other~~ as the others when q fluctuates slightly, which indicates that the optimal solution is robust to minor fluctuations ~~of~~ in q . Therefore, ~~even~~ though the facility failure probability is not easy to estimate precisely, the optimal solutions seem insensitive to minor fluctuations ~~of~~ in the facility failure probability.

In summary, when making ~~the~~ location and protection decisions, the decision makers should take into account the facility failure probability, demand and ~~the~~ geographical position into account. Generally, when the failure probability is high, managers should ~~better~~ open less unreliable facilities and open more reliable facilities to reduce the rerouting cost, and vice versa. At the same time, it seems better to centralize ~~on protecting~~ the protection of a few large cities or transportation hubs,

Table 2. The sites with facilities opened corresponding to different q.

q	sites with unreliable facilities opened	sites with reliable facilities opened
0.01-0.02	8,13,24,32,46,50,53,55,57,65,68,83,89,95,97	0,20,27,39
0.03-0.04	8,13,24,32,46,50,53,55,57,65,68,83,89,95,97	0,20,23,39
0.05-0.08	8,13,24,32,50,55,57,65,68,83,89,95,97	0,20,23,42
0.09-0.11	8,13,24,32,50,55,57,65,68,83,95,97	0,20,27,42
0.12-0.13	8,13,24,32,50,55,65,68,83,95,97	0,20,27,42
0.14-0.17	13,20,24,32,50,55,65,68,83,95,97	0,15,27,42
0.18-0.2	13,20,24,32,50,55,65,68,83,95,97	0,15,27,39,

~~since~~ as these cities appear in the optimal solutions most frequently. Finally, the fixed cost seems insensitive to the facility failure probability~~,~~ ; thus, even when the budget for fixed ~~charge~~ charges is limited, decision makers can still deal with high ~~disruptions risk through~~ disruption risks by adjusting the number of reliable and unreliable facilities.

Impact of the protection cost. ~~Since~~ Because $f_j^R = f_j^U + W \cdot q_j$, the ~~protecting~~ protection cost is not only related to the failure probability ~~, but also related to the protecting~~ but also to the protection cost coefficient W, which represents the cost for reducing the unit failure probability. The coefficient W is unrelated to the failure probability, but it is related to some other factors, such as the technical factors. Therefore, it may be relatively low under some circumstances but much higher under other circumstances. In this section, we discuss how the fluctuations of W affect the location and protection decisions.

We let ~~the~~ W vary from 1,000,000 to 12,500,000 and then observe its impact on the location decisions and the overall cost. At the same time, we let $q_j = 0.025$ and $q_j = 0.125$ ~~, respectively,~~ to represent the cases when the failure probability is low and high, ~~and still,~~ respectively, and we let $f_j^R = f_j^U + 5,000,000q_j$. We record the number of opened facilities and different terms of cost corresponding to different W for ~~the~~ both cases, which are depicted in Fig. **??** and Fig. **??**.

Figure 6. Number of sites with facilities opened corresponding to different W.

Figure 7. ~~Cost~~ Costs corresponding to different W and two failure probabilities.

As can be seen from Fig. **??**, the numbers of both reliable and unreliable facilities are affected remarkably by W. As W ~~becomes larger~~increases, which means that protection becomes more expensive, less reliable facilities are opened~~, and~~ ; at the same time, more unreliable facilities are opened~~, this~~ . This trend is especially obvious when W increases from 1,000,000 to 5,000,000. These observations are in ~~accord with our intuitions,~~ accordance with our intuition: when the protection price is high, we should open less reliable facilities while opening more unreliable facilities, and vice versa.

Now, we observe the impact of W for different facility failure ~~probability~~ probabilities q. As can be seem from Fig. **??**, the numbers of reliable facilities when $q = 0.025$ and when $q = 0.125$ are very ~~closed to each other. Actually, they~~ close in value. They are the same for ~~all~~ the range of W, ~~only except for the case~~ except when $W = 10,000,000$. ~~By the contrary~~In contrast, the number of unreliable facilities when $q = 0.125$ is much smaller than that when $q = 0.025$. ~~These indicate~~ This indicates

that the number of reliable facilities is mainly affected by W, but the number of reliable facilities is affected by both W and q.

As can be seen from Fig. ??, both the overall cost and the service cost increase as W increases, and this trend is more obvious when the failure probability is high. More specifically, when $q = 0.025$, both the total cost and the service cost increase only ~~lightly~~ slightly with W. For example, when W increases from 1,000,000 to 12,500,000, the total cost increases by 6.07% and the transportation cost increases by 0.23%. However, when $q = 0.125$, the objective value increases by 28.73% and the transportation cost increases by 35.17%. Therefore, if the failure probability is high, managers should try different ways ~~to reduce~~ of reducing the protection cost coefficient W, ~~since~~ as it can impact both the service cost and total cost significantly~~,~~ ; otherwise, they should not worry too much about a ~~big~~ large W.

Compared to the service cost, the fixed cost seems much less sensitive to W, which holds true for ~~the both cases~~ when q=0.025 and q=0.125. More specifically, as W increases from 1,000,000 to 12,500,000, i.e., it becomes 12.5 times ~~bigger~~larger, the fixed cost increases by only 14.32% when q=0.025 ~~, and increases~~ and increases by only 19.13% when q=0.125. The insensitivity of the fixed cost to W indicates that when the protection measures are expensive, decision makers can still obtain ~~low fixed cost solution~~low-fixed-cost solutions, which can be ~~done through reducing reliable facilities~~ achieved by reducing the number of reliable facilities opened and opening more unreliable facilities.

Impact of the emergency supply cost. The emergency supply cost coefficient d^B_{ij}/d^P_{ij} is the ratio ~~between~~ of the emergency supply cost ~~and~~ to the regular cost for each unit demand, which reflects the system's capability ~~to cope with the facility disruptions, with a lower value denoting~~ for coping with facility disruptions. A lower value denotes a better reactive emergency mechanism. The special case when $d^B_{ij}/d^P_{ij} = 1$ represents the ideal situation, namely, the emergency cost is the same as the regular cost. However, this ideal situation may not be easy to ensure in reality, and ~~always we~~ we always have $d^B_{ij}/d^P_{ij} > 1$. In this section, we discuss the impact of the emergency supply cost coefficient on the optimal solution and different terms of cost.

We let d^B_{ij}/d^P_{ij} increase from 1 to 2 ~~,~~to model the cases when the emergency supply becomes more and more expensive, and we set $q = 0.025$ and $q = 0.125$ ~~respectively,~~ to represent the cases when the facility failure risk is low and high~~. The cost~~ , respectively. The costs of different terms and the ~~number~~ numbers of opened facilities according to different d^B_{ij}/d^P_{ij} are ~~depicted~~ presented in Fig. ?? and Fig. ??.

Figure 8. ~~Cost~~ Costs of different terms corresponding to different d^B_{ij}/d^P_{ij}.

Figure 9. ~~Number~~ Numbers of opened facilities corresponding to different d^B_{ij}/d^P_{ij}.

~~Intuitionally~~Intuitively, one may think that the emergency supply cost coefficient affects the total cost significantly. ~~Since the higher the~~ Because the higher d^B_{ij}/d^P_{ij} is, the more expensive ~~to get~~ it is to obtain an emergency supply, especially when the facility failure probability is high, the emergency supply will be triggered more frequently, thus resulting in higher emergency service ~~cost~~costs. However, as can be seen from Fig. ??, the objective value increases rather slowly as ~~the~~ d^B_{ij}/d^P_{ij} increases, ~~for~~ both when q=0.025 and q=0.125. More specifically, when d^B_{ij}/d^P_{ij} becomes ~~2 times larger~~twice as large, the objective value increases by only 1.51% when q=0.025 ~~, and increases~~ and increases by only 8.87% when q=0.125.

Therefore, the overall cost is actually insensitive to d_{ij}^B/d_{ij}^P, at least when $q \leq 0.125$. This seems counterintuitive at ~~the~~ first glance, but ~~it~~ can be explained easily. When d_{ij}^B/d_{ij}^P ~~becomes larger~~increases, the only increasing cost is the emergency service cost, which is a part of the total service cost and also a small part of the total cost. Even when d_{ij}^B/d_{ij}^P ~~becomes much larger, say~~ increases greatly, say to 2, and even when the facility failure probability is relatively high, say 0.125, ~~and assume~~ assuming that the locations are the same, the increasing ratio of the emergency cost is still only $2 * 0.125 = 0.25$, and the increasing ratio of the total cost is much smaller than 0.25 ~~since~~ as the emergency cost is only part of it. This property is very useful, ~~it reminders~~ as it reminds the decision makers ~~of~~ that even when the emergency cost for each unit demand is high, they can still obtain cost-effective ~~solution through determining~~ solutions by setting the number of both unreliable and reliable facilities wisely, ~~because~~ as the objective value is actually insensitive to the emergency cost.

Fig. **??** ~~shows~~ presents the number of opened facilities corresponding to different d_{ij}^B/d_{ij}^P. It can be easily seen that the number of unreliable facilities ~~drops~~ decreases and the number of reliable facilities increases as d_{ij}^B/d_{ij}^P ~~becoming larger~~increases. This trend is more obvious when the facility failure probability is high. Therefore, when the emergency supply cost is high, the decision makers should open more reliable and less unreliable facilities to reduce ~~its impact. Through~~ the impact. By protecting more facilities, the overall cost will not be affected too much when the emergency service cost ~~becomes higher~~increases. However, the decision makers should also take the protection cost into account and make good ~~trad-offs~~trade-offs.

Conclusions

This paper proposes an integer programming model for the reliable facility location problem with facility protection, which allows for site-specific failure probabilities. In the model, both the proactive measure, i.e., protecting some of the facilities, and the reactive measure, i.e., getting emergency supply from a backup facility when a customer's primary facility fails, are used. This facility protection and ~~single level~~ single-level backup mechanism can increase ~~the~~ facility availability while reducing the operating complexities of ~~a~~ facility networks.

An effective solution approach ~~which~~ that combines Lagrangian Relaxation and local search is developed to solve the model. ~~Through~~ Using numerical examples with different sizes, i.e., networks with 49, 88, 150 and 263 nodes~~respectively, the performance~~ , the performances of the proposed algorithms are compared with CPLEX, and the computational results show that our approach works well and consumes much less CPU time than CPLEX ~~for all~~ does for all of the examples.

~~Through~~ Using a practical example ~~in~~ from China, the influences of the facility disruption probabilities, protection cost and emergency service cost are analyzed, and we find that the overall cost ~~increase~~ increases as the facility failure probability and protection cost increase, but it is insensitive to the emergency service cost. ~~The decision~~ Decision makers should wisely determine the number of both reliable and unreliable facilities to obtain a ~~cost-efficient~~ cost-effective solution.

In this work, all of the facilities are assumed to be uncapacitated, and a meaningful extension ~~is~~ would be to consider the facility capacity constraints, which can make the problem reflect more practical situations. ~~Also~~Additionally, as stated before, the facility failure probabilities are not easy to estimate precisely~~,~~; thus, considering ~~the~~ facility protection and backup assignment strategies in other modeling frameworks, such as robust optimization~~etc.~~, may be a promising future direction. Finally, designing a facility network ~~which~~ that is robust to both random disruptions and deliberate attacks is also worthy ~~for further studies~~of further study.

Supporting Information

S1 Dataset

The computational experiment dataset. ~~Including~~ Includes the 49,88,150 and 263 nodes datasets, each dataset contains 20 randomly generated instances.

The case example dataset. ~~Including the information about~~ Includes the information on the Hunan case example.

Acknowledgments

This research was supported by the National Science Foundation of China Grant (61273322,71571186,71201169). The research of the first author was also supported by the China Scholarship Council (20123013). All ~~the supports are~~ support is gratefully acknowledged.
